# *PATL2* mutations affect human oocyte maternal mRNA homeostasis and protein interactions in cell cycle regulation

**DOI:** 10.1186/s13578-024-01341-2

**Published:** 2024-12-31

**Authors:** Yin-Li Zhang, Zhanhong Hu, Huifang Jiang, Jiamin Jin, Yan Zhou, Mengru Lai, Peipei Ren, Siya Liu, Ying-Yi Zhang, Yan Rong, Wei Zheng, Shen Zhang, Xiaomei Tong, Songying Zhang

**Affiliations:** 1 Assisted Reproduction Unit, Department of Obstetrics and Gynecology, Sir Run Run Shaw Hospital, School of Medicine, Zhejiang University, Hangzhou, 310016 China; 2 Zhejiang Provincial Clinical Research Center for Reproductive Health Diseases, Hangzhou, 310016 China; 3 Zhejiang Key Laboratory of Precise Protection and Promotion of Fertility, Hangzhou, 310016 China; 4 Clinical Research Center for Reproduction and Genetics in Hunan Province, Reproductive and Genetic Hospital of CITIC-Xiangya, Changsha, 410078 China

**Keywords:** *PATL2* mutation, TUT7, CDC23, mRNA storage, mRNA decay

## Abstract

**Background:**

Oocyte maturation defect (OMD) and early embryonic arrest result in female infertility. Previous studies have linked biallelic mutations in the *PATL2* gene to OMD, yet the underlying mechanism remains largely unknown.

**Results:**

This study uncovers three novel mutations (c.1201G > T, c.1284delA and c.1613 + 2_1613 + 3insGT) and three reported mutations (c.1204 C > T, c.1271T > C, c.223 − 14_223-2delCCCTCCTGTTCCA) in the *PATL2* gene across five unrelated individuals exhibiting OMD, oocyte death, and early embryonic arrest. RNA sequencing revealed that *PATL2* mutations decreased mRNA storage in human germinal vesicle (GV) oocytes and impeded mRNA decay during maturation and in early embryos. We demonstrate that PATL2 interacts with CPEB1 and TUT7 in human oocytes to maintain mRNA homeostasis. Additionally, we observed a reduction in *CCNB1* and *CCNE1* mRNA levels in *PATL2*-mutant GV oocytes, which may be linked to GV arrest. Employing both wild-type and mutated PATL2^V401F/R402W^ variants, we characterized the protein interactome of PATL2, identifying disruptions of PATL2^V401F/R402W^ variants predominantly affecting cell cycle-related proteins, including CDC23, APC1 and MAD2L1. PATL2’s interaction with and stabilization of CDC23 in oocytes may elucidate the mechanisms behind the mutation-induced MI arrest. PALT2 is required for the efficient mRNA translation and it maintains the protein level of CDC23, APC1 and MAD2L1 in mouse GV oocyte.

**Conclusion:**

PATL2 plays a critical role in regulating mRNA accumulation and decay in human oocytes, potentially through interactions with CPEB1 and TUT7, respectively. Mutations in PATL2 lead to oocyte meiosis defects by affecting the mRNA accumulation, mRNA translation, and direct binding to and stabilizing proteins related to cell cycle regulation, such as CCNB1 and CDC23. This study expands the mutational spectrum of *PATL2* and provides new insights into the molecular mechanisms underlying *PATL2* mutation-associated oocyte maturation disorders.

**Supplementary Information:**

The online version contains supplementary material available at 10.1186/s13578-024-01341-2.

## Background

The widespread application of assisted reproductive technology (ART) provides a unique opportunity to examine oocyte quality and maturation, pivotal for successful fertilization and early embryonic development [1, 2]. In clinical practices, ART employs morphological assessments to categorize oocyte meiotic stages or maturity—GV (germinal vesicle), MI (metaphase I), and MII (metaphase II). The transition of an oocyte to the MII stage indicates maturity and readiness for fertilization. Both meiotic progression and cytoplasmic maturation are crucial, as defects in maternal mRNA accumulation or abnormal mRNA clearance leads to oocyte meiosis defect or early embryonic arrest [3–5].

The precise orchestration of meiotic progression is critically dependent on the stringent regulation of maternal mRNA accumulation and protein synthesis within maturing oocytes [6, 7]. Central to this regulation is the maturation promoting factor (MPF) [8], a complex consisting of the catalytic subunit CDK1 (also known as Cdc2) and its regulatory partners, the cyclin B isoforms (CCNB1, CCNB2, and CCNB3) [8, 9]. CDK1 remains inactive while oocytes are in the GV stage, awaiting cues for meiotic resumption. The accumulation of cyclin B mRNA and protein is necessary for re-entry into meiosis [10], and the precise balance of cyclin B protein synthesis and degradation is pivotal for advancing through meiotic stages. Cyclin B protein is degraded by the anaphase-promoting complex/cyclosome (APC/C) [11], a conserved E3 ubiquitin ligase playing a key role in the metaphase/anaphase transition during both mitotic and meiotic cycles across various species [12, 13]. The APC/C, composed of 19 subunits organized into scaffold [14], catalytic, tetratricopeptide repeat, and substrate recognition modules, is integral to oocyte meiosis. Critical subunits, such as CDC20 [15], CDH1 [16], and APC8 (also referred to as CDC23) [17, 18], are required for oocyte meiosis, highlight the complex’s essential role in ensuring proper meiotic progression.

Oocyte maturation defect (OMD) causes female infertility and ART failure, characterized by the interruption of the oocyte meiotic cycle at various stages: GV, MI, MII, or mixed arrest [19]. Several genes, including *TUBB8* (MIM: 616768) [20], *TRIP13* (MIM: 604507) [21], *PATL2* (MIM: 614661) [22], *TBPL2* (MIM: 608964) [23], *CDC20* (MIM: 603618) [24], *MOS* (MIM: 620383) [25], *CDC23* (MIM: 603462) [17], and *PABPC1L* (NM_001372179) [26], have been linked to OMD, with *PATL2* mutations commonly found in patients experiencing GV or MI arrest [22, 27, 28]. Notably, PATL2 encodes an RNA-binding protein that was initially identified as a translational repressor in Xenopus oocytes [29, 30], and the overexpression of PATL2 ortholog (labeled P100) represses mRNA translation and blocks oocyte meiosis I progression [30]. *PATL2* loss-of-function mutations in humans lead to GV and MI arrest [22], but PATL2 knockout mice exhibit mild OMD phenotypes, mainly exhibiting early embryo arrest phenotype [31, 32]. The *Patl2*-null mouse model revealed that PATL2 mainly functions as an adaptor protein to recruit EIF4E and CPEB1 to maintain the mRNA stability and maternal transcripts dosage in growing oocytes [31]. This complex interplay ensures proper mRNA translation post-GVBD, triggered by PATL2 phosphorylation and subsequent protein degradation [31]. The exact pathogenic mechanism by which PATL2 regulating human oocyte meiosis is yet unknown.

In this study, three novel pathogenic variants in *PATL2* are identified in infertile women exhibiting recurrent OMD in first part, and we validate the pathogenic effects of mutations on PATL2 protein and its function. In the second part, we found the protein interactome alteration between PATL2 and PATL2 ^V401F/R402W^ mutants, and demonstrate that PATL2 directly binds to some key cell cycle-related proteins, such as CDC23, APC1 and MAD2L1. These PATL2 mutations result in decreased protein-protein interactions and compromised protein stabilization, particularly with CDC23. In the third part, we explore the role of PATL2 in mRNA regulation using RNA-seq of human oocytes and embryos with *PATL2* mutations and investigate potential mechanism. Finally, we detect the function of PATL2 on mRNA translation. This study demonstrates that PATL2 mutations induce OMD through affecting mRNA and protein level of cell cycle-related proteins. Our findings establish the causal relationship between *PATL2* and the phenotype of multi-phenotype in human, which may provide new understanding of PATL2 for female infertility.

## Materials and methods

### Clinical samples

We recruited infertile individuals diagnosed with OMD from the Sir Run Run Shaw Hospital. All blood samples, oocytes and early embryos were obtained for investigation after informed consent. Studies of human subjects were approved by the Ethics Committee of Sir Run Run Shaw Hospital (NO. 20220461).

### Animals

Wild-type ICR female mice (3-weeks old) were purchased from the Zhejiang Academy of Medical Sciences (Hangzhou, China). These mice were maintained under specific pathogen-free (SPF) conditions. All animal experiments were performed in accordance with the guidelines of the Animal Committee of Zhejiang University.

### Whole-exome sequencing and *PATL2* variant screening

Blood samples from patients exhibiting OMD were subjected to DNA extraction and whole-exome sequencing. *PATL2* variants and the known genes responsible for OMD and female infertility were filtered. Functional prediction was assessed using the SIFT and Polyphen programs.

### Sanger sequencing

Specific primers flanking the variants in the *PATL2* gene were used for amplification by PCR, followed by Sanger sequencing analysis using ABI 3100 DNA Analyzer in Tsingke Biotech Co., Ltd. (Hangzhou, China). The primes were listed in supplementary Table 1.

### Molecular modeling and evolutionary conservation analysis

Evolutionary conservation analysis was performed using the online UniProt software. The three-dimensional structure of wild-type PATL2 (NP_001138584.1, UniprotKB ID: C9JE40) was predicted using the Alphafold2. Molecular graphics and analysis were performed using the Schrödinger software. Structural stability analysis was performed using the Imutant2.0 software to calculate the free energy of the protein (ΔΔ G) to predict the impact of protein mutations on structural stability.

### Plasmids

The pCMV3-PATL2 (HG26025-UT) with full-length human *PATL2* cDNA (NM_001145112.2) was obtained from Sino Biological Inc. (Beijing, China). After sequencing, pCMV3-PATL2 was used as a template for mutagenesis using the QuikChange II site-directed mutagenesis kit (200524, Agilent Technologies, Santa Clara, CA, USA). FLAG-labeled and HA-tagged PATL2 plasmids were generated using ClonExpress Ultra One Step Cloning Kit (C115-01, Vazyme, Nanjing, China) according to the manufacturer’s instructions. All plasmids were verified by Sanger sequencing prior to transfection. Flag-CDC23 plasmids was reported previously [17] and was gifted by Zhihua Zhang in professor Lei Wang group.

### Cell culture and plasmid transfection

Human HEK293T cells were cultured in DMEM medium (Gibco, Waltham, MA, USA) supplemented with 10% fetal bovine serum (FBS, CellMax), 100 U/mL penicillin, and 100 µg/mL streptomycin at 37 °C in 5% CO_2_. When cells reached 50–70% confluence, the same amounts of plasmids of *PATL2* variants were transfected using Polyethylenimine Linear (PEI) (40816ES02, Yeasen) according to the manufacturer’s instructions. Approximately 48 h after transfection, cells were fixed for immunofluorescence, or performed co-immunoprecipitation and western blotting.

### Oocyte collection and culture

The 3–4 weeks old female mice were injected with 5 IU pregnant mare serum gonadotropin (PMSG, Ningbo San Sheng Biotech, Ningbo, China) and humanly killed 44 h later. GV oocytes were collected by puncturing the large ovarian follicles in M2 medium (Sigma, M5910). For microinjection, the GV oocytes were cultured in M2 medium with 2.5 µM milrinone (HY14252, MCE). The GV oocytes were matured in vitro to MII stage in M2 medium for 14 h.

### In vitro mRNA transcription and microinjection

The cRNAs of HA-PATL2 variants were in vitro transcribed as previously described [33]. Briefly, HA-tagged PATL2 plasmids were liberalized using *Kpn1* restriction enzymes. 5’-capped mRNAs were transcribed using mMESSAGE mMACHINE T7 Ultra Transcription Kits (AM1345, Invitrogen, Carlsbad, CA, USA). Synthesized *PATL2* cRNAs with poly(A) were recovered with lithium chloride precipitation at -20 °C, cleared with 70% ethanol, and finally, dissolved in nuclease-free water. All microinjections were performed using a Narishige micromanipulator. Approximately 10 pL of cRNAs (∼300 ng/µL) were microinjected into the ooplasm of GV oocytes. GV oocytes were cultured in M2 medium with 2.5 µM milrinone (HY14252, MCE) for 24 h for cRNA translation, prior to being released into fresh M2 medium.

### Immunofluorescence

Mouse oocytes were fixed in 3.7% paraformaldehyde diluted in PBS for 30 min at room temperature and then permeabilized with 0.2% TritonX-100 for 15 min. After incubation for 1 h in blocking buffer (1% BSA diluted in PBS with 0.1% TritonX-100), oocytes were stained with the indicated primary antibodies diluted in blocking buffer overnight at 4 °C. After three washes, samples were incubated with Alexa Fluor 568-conjugated goat anti-rabbit (A11036, Invitrogen), Alexa Fluor 488-conjugated donkey anti-mouse (A10037, Invitrogen) secondary antibodies, in combination with DAPI (236276, Roche, Basel, Switzerland), for 1 h at room temperature. After washing four times, the oocytes were mounted on slides with antifade medium and imaged using a laser-scanning confocal microscope (LSM800, Carl Zeiss, Jena, Germany). The primary antibodies used were as follows: rabbit polyclonal anti-PATL2 (bs-19898R, Bioss Antibodies, China), mouse monoclonal anti-FLAG (F1804, Sigma-Aldrich), rabbit anti-HA tag (3724, Cell Signaling Technology), and rabbit monoclonal anti-CDC23 (AF2716, Beyotime Biotechnology, China).

### Detection of protein synthesis

To detect new protein synthesis, GV oocytes were cultured in M2 medium supplemented with 1% BSA (Sigma Aldrich), 2.5 μm milrinone and 100 µM HPG for 2 h. Then oocytes were fixed in 3.7% PFA. HPG was detected using a Click-iT HPG Alexa Fluor Protein Synthesis Assay Kit (Life Technologies) according to the manufacturer’s instructions.

### Western blotting

Cells transiently expressing the indicated plasmids were lysed using 1x Laemmli sample buffer (1610747, Bio-Rad). Proteins were separated via SDS-PAGE and transferred onto a polyvinylidene difluoride (PVDF) membrane. After blocking in TBS containing 5% defat milk for 1 h at room temperature, membranes were incubated with primary antibodies overnight at 4 °C. After washing three times in TBS-Tween-20 (0.05%), membranes were incubated with goat anti-rabbit horseradish peroxidase-conjugated (1:5,000, 111-035-003, Jackson ImmunoResearch, West Grove, PA, USA) or goat anti-mouse (1:5,000, 115-035-003, Jackson ImmunoResearch) secondary antibodies for 1 h at room temperature. Signals were detected using enhanced chemiluminescence (Millipore, Burlington, MA, USA). The primary antibodies used were as follows: mouse monoclonal anti-FLAG (F1804, Sigma-Aldrich), rabbit anti-HA tag (3724, Cell Signaling Technology), rabbit polyclonal anti-APC1 (21748-1-AP, Proteintech), rabbit monoclonal anti-CDC23 (AF2716, Beyotime Biotechnology, China), mouse monoclonal anti-GAPDH (60004-1-Ig, Proteintech), rabbit anti-MAD2L1 (10337, Proteintech), and rabbit polyclonal TUT7 (25196, Proteintech).

### Co-immunoprecipitation

Immunoprecipitation assays were performed as previously described [34]. Briefly, after transfection of the indicated plasmids for 36 h, cells were washed with cold PBS and then lysed using lysis buffer [freshly added proteinase inhibitor cocktail (Roche) and PMSF (Beyotime)] at 4 °C for 30 min. Subsequently, FLAG-M2 magnetic beads (M8823, Sigma-Aldrich) or EZ veiw anti-HA affinity gel (E6779, Sigma-Aldrich) were prepared and added to the cell extracts and incubated overnight at 4 °C. Beads were washed four to five times with lysis buffer, and the immune complexes were subjected to western blotting.

### Minigene splicing assay

The wild-type and splicing mutant DNA fragments (1496 bp), which were generated by nested PCR, were inserted into pcDNA3.1(+) plasmid. The wild type and mutant plasmids were transfected into HeLa or 293T cells for 36 h, followed by mRNA extraction using Trizol Reagent (Invitrogen). The mRNAs were subjected to first strand reverse transcription (Vazyme), and then the cDNAs were used for PCR amplification (Vazyme). The amplified DNA fragments from wild type or splicing mutant group were performed Sanger Sequencing using ABI 3100 DNA Analyzer in Tsingke Biotech Co., Ltd. (Hangzhou, China).

### Mass spectrometry of Co-IP eluents and data analysis

HA vector, HA-PATL2 and HA-PATL2^V401F/W402F^ plasmids were transfected into 293T cells for 48 h. The co-IP was performed using Esay view HA affinity beads (E6779, Sigma) as described above. After three times washing with IP lysis buffer with 250 mM NaCl, the beads-immunoprecipitants were washed by ultrapure water for 3 times, followed by washing 5 times using the 50 mM TEAB (Sigma-Aldrich, T7408). Then, 35 µl 50 mM TEAB was added into samples, followed by the addition of 5 µL of 0.05 µg/µL trypsin (Promega, V5280). The samples were digested in 37 °C for 4 h with shaking at 400 rpm. The supernant was transferred into 200 ul EP tube by addition with 5 µL of 0.05 µg/µL trypsin and was subjected to further digestion for overnight in 37 °C as described previously [35]. Then, 1 µL formic acid was added. The digested peptides were loaded for MS analyses using a Thermo Fisher Orbitrap Eclipse Tribrid mass spectrometer as reported previously [36]. Proteome Discoverer Software (version 2.5, San Jose, CA) was used to process raw files for detecting features, searching databases and quantifying proteins/peptides.

The search of MS/MS spectra was conducted against the UniProt human database (downloaded on June 27th, 2022, containing 79,435 entries). Methionine oxidation and N-terminal protein acetylation were chosen as variable modifications, while the carbamidomethylation of cysteine residues was regarded as a fixed modification. Precursors and fragments had a mass tolerance of 10 ppm and 0.6 Da, respectively. Minimum and maximum peptide lengths were six and 144 amino acids, respectively. The missed cleavage allowed for every peptide was two. The filtering of proteins had a maximum false discovery rate (FDR) of 0.01. Proteins with |log2 FC| > log2 [10] was considered DEPs in [PATL2 vs. Vector] and [PATL2^V401F/R402W^ vs. Vector] and proteins with |log2 FC| > log2(2/3) was considered DEPs in [PATL2^V401F/R402W^ vs. PATL2]. A summary of proteomics data generated in this study is shown in Supplementary Table S4.

### Single oocyte or embryo RNA sequencing (RNA-seq)

Human GV and MII oocytes were donated from unidentified control patients and patients with the MOS^Asn95Lys^ homozygous variant. A single oocyte was lysed using 4 µL cell lysis buffer, and cDNA was obtained via reverse transcription using the SMART-seq2 method, as previously described [37]. In brief, first-strand cDNA synthesis and amplification were performed using SuperScript II (18064014, Thermo Fisher Scientific) and KAPA Hotstart Hifi ready mix (KK2601, Roche). Sequencing libraries were constructed using TruePrep DNA Library Prep Kit V2 for Illumina (TD503, Vazyme Biotech, Nanjing, China), followed by sequencing on an Illumina HiSeq 2500 with 150-bp long paired-end reads.

### RNA-seq data analysis

RNA-seq data were processed using standard procedures as previously described. In brief, the raw transcriptome sequencing data were trimmed using Trim Galore (version 0.6.10) and Cutadapt (version 1.18) to remove primer sequences. The clean reads were then mapped to the human reference genome of GRCh38 using Hisat2 (v2.2.1). Symbol genes were calculated using featureCounts (v2.0.6). The expression levels of each gene were quantified using normalized TPM (Transcripts Per Kilobase per Million mapped reads) and were further normalized with the ERCC spike-in. The differential gene expression was analyzed with the R package DESeq2. Genes with |log2 FC| > 1 and adjusted *P* value (by the Benjamini–Hochberg method) < 0.05 were considered DEGs. GO and KEGG enrichment analysis of the DEGs were performed using the clusterprofiler package in R. The transcript clustering was described previously [5]. Transcripts with reliable sequence annotations and TPM of > 2 in control group were used for further analysis. Expression levels of each gene were added to one and then transformed by log2 in the following analysis with a 2-fold as a differential expression.

### Image acquisition and quantification

For bright-field image acquisition of oocytes and embryos, a Nikon Ts2R microscope with a Hoffman system was used. Fluorescent images were captured using an LSM800 laser scanning confocal microscope (Carl Zeiss). ImageJ software was used for signal quantification.

### Statistical analysis

Statistical analysis was conducted using the GraphPad Prism 7.0 software (GraphPad Software Inc., La Jolla, CA, USA). The data are expressed as mean ± standard error (SD). Differences between two groups were compared using the Student’s *t*-test. In the case of more than two groups, one-way ANOVA was used, followed by *post hoc* Tukey’s test for multiple comparisons. *P* < 0.05 was considered statistically significant.

## Results

### Clinical characteristics of the affected individuals

All patients presented with primary infertility of undetermined etiology, having undergone several unsuccessful in vitro fertilization-embryo transfer (IVF-ET) cycles. In family 1, the individual II-1 endured eight years of primary infertility and underwent three oocyte retrieval attempts. Unfortunately, all 65 oocytes retrieved were unsuitable for IVF or ICSI due to immaturity (GV or MI stages) or morphological abnormalities (Fig. 2A), as detailed in Table 1. Notably, during the third retrieval, in vitro maturation (IVM) was performed for nine MI-stage oocytes, resulting in four oocytes exhibiting two-cell-like morphology, suggestive of large polar bodies (Fig. 2A).

**Table 1:** Oocyte characteristics of IVF and ICSI attempts for the affected individuals

Family NO.	Age (Years)	Duration of Infertility (Years)	IVF or ICSI Attempt	Retrieved Oocytes	GV Oocytes	MI Oocyte	MII Oocyte	Abnormal Morphology Oocyte	Fertilized Outcomes (2PN)
1	32	8	IVF	29	12	13	0	4 ^a^	0
			IVF	17	8	0	0	9 ^a^	0
			N/A	19	8	9	0	2	0
2	28	5	IVF	19	0	0	8	11 ^a^	0
			ICSI	12	2	2	6 (big PB1)	2	0 (3 0PN embryos, all arrested at 2–5 cells)
3	31	6	ICSI	3	0	0	3	0	2 (1 0PN embryos, all arrested at 2–4 cells)
			N/A	2	0	1	0	1	0
			N/A	1	0	0	0	1	0
4	35	10	IVF	10	2	1	7	0 ^a^	0
			ICSI	11	2	2	7	0 ^a^	3 (4-cell Arrest)
			ICSI	9	2	1	6	0 ^a^	2 (4-cell Arrest)
			ICSI	9	1	1	7	0	4 (2-5-cell Arrest)
5	35	7	IVF ICSI	12 14	6 3	2 0	0 2 (big PB1)	4 9 (7 degeneration)	0 0

^a^ The patient dictated the extra attempts at other hospitals

Abbreviation: IVF, in vitro fertilization; ICSI, intracytoplasmic sperm injection; GV, geminal vesicle; MI, metaphase I; MII, metaphase II; PB1, the first polar body; PN, pronucleus; N/A, not available

In family 2, proband II-1, after five years of diagnosed primary infertility, underwent two ICSI attempts. From 31 retrieved oocytes, only 14 were mature (MII stage), while four remained immature and the rest were abnormal. This cycle resulted in three 3PN zygotes, which consistently arrested at the 2- to 5-cell stage, as shown in Table 1; Fig. 2A.

In family 3, patient II-1 underwent a single IVF cycle, yielding two 2PN zygotes and one 0PN zygote; however, all embryos arrested at 2- to 4-cell stage. Subsequent attempts yielded only three immature or abnormal oocytes (Table 1).

Family 4’s patient II-1 displayed a higher proportion of mature oocytes (27 MII out of 39 retrieved), yet the remaining oocytes were arrested at either the GV or MI stage. Of the nine zygotes obtained, all embryos ceased development at the 2- to 5-cell stage (Table 1; Fig. 2A).

Lastly, in family 5, patient II-1 from a consanguineous background underwent two IVF/ICSI cycles, retrieving 26 oocytes. Of these, 9 were arrested at the GV stage, 2 at the MI stage, with the remainder exhibiting morphological abnormalities or degeneration (Fig. 2A). Detailed clinical characteristics of the retrieved oocytes are summarized in Table 1.

### Identification of novel *PATL2* variants in oocyte maturation defect patients

**Fig. 1:**
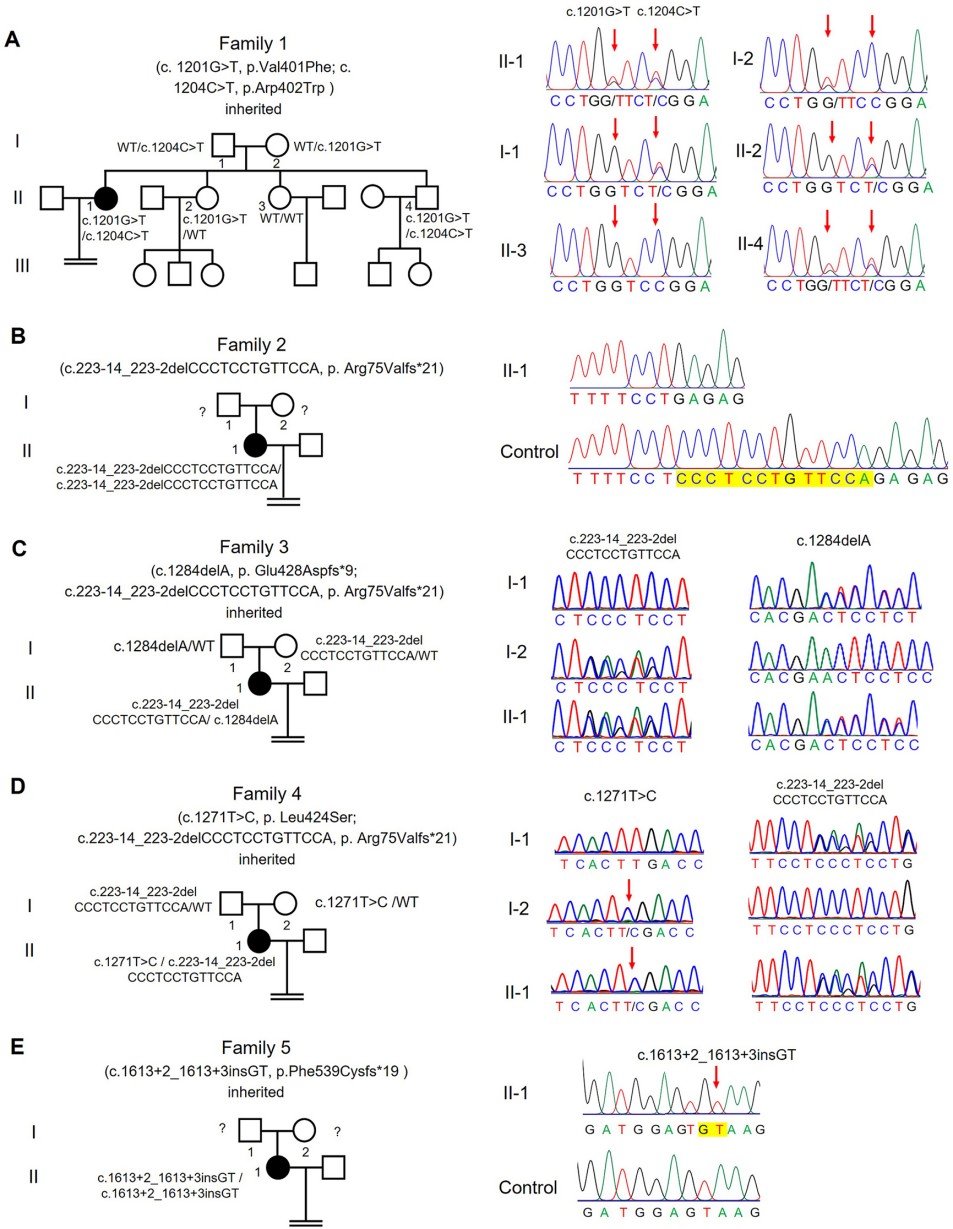
Identification of *PATL2* gene mutations in five unrelated infertile individuals. **(A-E)** Pedigrees of the 5 families affected by female infertility with Sanger sequencing confirmation below. Squares denote male family members, circles denote female members, solid circles denote affected individuals, and equals signs represent infertility. Red arrows highlight the mutation sites

Whole-exome sequencing (WES) was employed to identify pathogenic variants in patients exhibiting oocyte maturation defects (OMD). Through this analysis, we identified three novel missense mutations, one novel frameshift mutation, one novel splicing mutation, and one previously reported splicing mutation in the *PATL2* gene. In family 1, the affected individual carried biallelic missense mutations: c.1201G > T (p.V401F) and c.1204 C > T (p.R402W), inherited from their father and mother, respectively, as depicted in Fig. 1A. Her two sisters, carrying either heterozygous or wild-type variants of *PATL2*, exhibited normal fertility. In family 2, the patient harbored a previously reported homozygous splicing mutation c.223 − 14_223-2delCCCTCCTGTTCCA (p.R75Vfs*21), with no available parental inheritance data (Fig. 1B). Family 3’s patient possessed compound heterozygous frameshift mutations: c.1284delA (p.E428Dfs*9) and c.223 − 14_223-2delCCCTCCTGTTCCA (p.R75Vfs*21), inherited from her mother and father, respectively (Fig. 1C). Similarly, the patient in family 4 exhibited compound heterozygous mutations c.1271T > C (p.L424S) and c.223 − 14_223-2del (p.R75Vfs*21), following the parental inheritance pattern (Fig. 1D). The individual from family 5 carried a homozygous splicing mutation c.1613 + 2_1613 + 3insGT with unknown inheritance data (Fig. 1E), verified to induce a frameshift (p.Phe539Cysfs*19) using mini gene assay (Figure S1A-E).

**Fig. 2:**
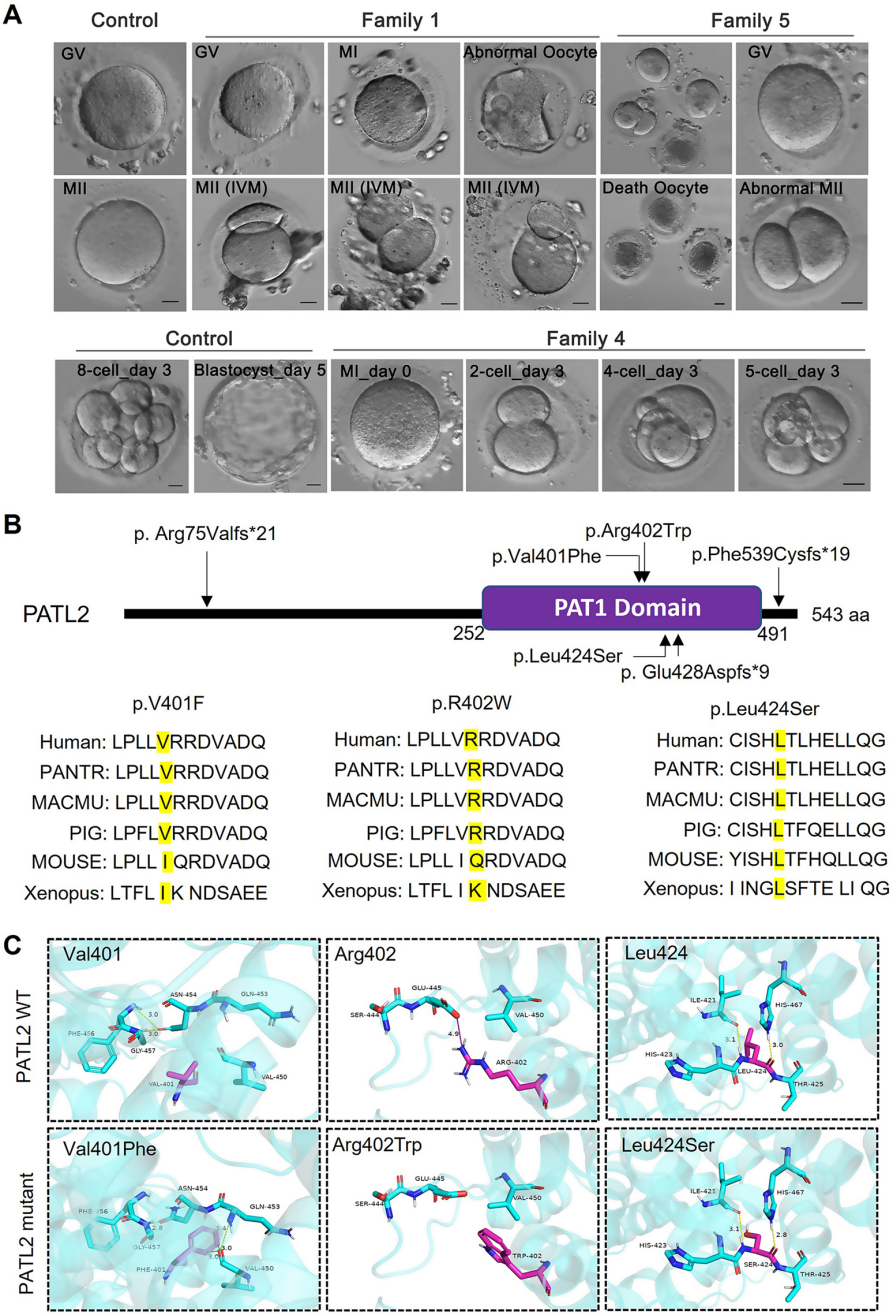
Effects of *PATL2* gene mutations on oocyte and embryo development and protein structure in female infertile patients (**A**) Bright field images showing the developmental stages of oocytes and early embryos from control individuals and patients from Families 1, 4, and 5 with *PATL2* mutations. Images include germinal vesicle (GV) oocytes, metaphase I (MI), and metaphase II (MII) oocytes, as well as various stages of embryos, such as the 8-cell stage and blastocyst formation in control, and 2-cell, 4-cell, and 5-cell stages in patients. Scale bars = 20 μm. (**B**) Schematic representation of the PATL2 protein and the distribution of PATL2 variants (the corresponding amino acid sequences) in PATL2 protein. The conservation of these mutation sites across different species (human, primates, mouse, pig, and Xenopus) with mutated residue marked in yellow. (**C**) *PATL2* missense variants encoding amino acid disrupted the ion pairs formed by wild-type PATL2 protein. The models compare the wild-type and mutant amino acid residues (Val401, Arg402, and Leu424) within the protein structure, illustrating how the mutations (Val401Phe, Arg402Trp, and Leu424Ser) alter the local conformation of the PATL2 protein

The pathogenic effects of three novel variants (the missense mutation p.V401F, the frameshift mutation p.E428Dfs*9, and the splicing mutation p.F539Cfs*19) and two newly identified variants (p.R402W and p.L424S) in a recently reported study [38] were not validated. The novel variants are absent from public databases (Table 2), indicating exceedingly low frequency. They are situated within the highly conserved PAT1 domain of PATL2, spanning species from *Xenopus laevis* to *Homo sapiens* (Fig. 2B). The frequency of the reported mutation p.R75Vfs*21 was recorded at approximately 7.2 × 10^− 6^ mutation frequency in the gnomAD exome database (Table 2). Three-dimensional structural analysis of PATL2 suggested that the missense mutations (V401F, p.R402W, and p.L424S) potentially disrupt hydrogen bonding, thereby destabilizing the protein (Fig. 2C). Pathogenic predictions using tools like SIFT and PolyPhen classify these variants as possibly or probably damaging (Table 2). Collectively, these findings underscore the pivotal role of *PATL2* mutations as a genetic basis for human OMD.

**Table 2:** Overview of the *PATL2* mutations observed in the five families

Genomic Position on Chr15 (bp)	SNP	cDNA Change	Protein Change	Exon	Mutation Type	SIFTa	PPH2a	1KG_east	ExAC_east
44,961,198	rs994341629	c.C1204 > T	p.Arg402Trp	12	Missense	D	D	NA	NA
44,961,201	NA	c.G1201 > T	p.Val401Phe	12	Missense	D	P	NA	NA
44,966,430–44,966,442	rs751701388 ^a^	c.223 − 14_223-2delCCCTCCTGTTCCA	p. Arg75Valfs*21	IVS3	Splicing	NA	NA	NA	0.0017
44,960,621	NA	c.1284delA	p.Glu428Aspfs*9	13	Missense	NA	NA	NA	NA
44,960,634	NA	c.1271 C > T	p.Leu424Ser	13	Missense	P	D	NA	NA
44,958,587	NA	c.1613 + 2_1613 + 3insGT	p. Phe539Cysfs*19	IVS15	Splicing	NA	NA	NA	NA

^a^ The variants have been reported previously. Abbreviation: N/A, not available; D, Deleterious, T, Tolerance, P, Possibly damaging

### Pathogenic effects of *PATL2* variants in protein property

**Fig. 3:**
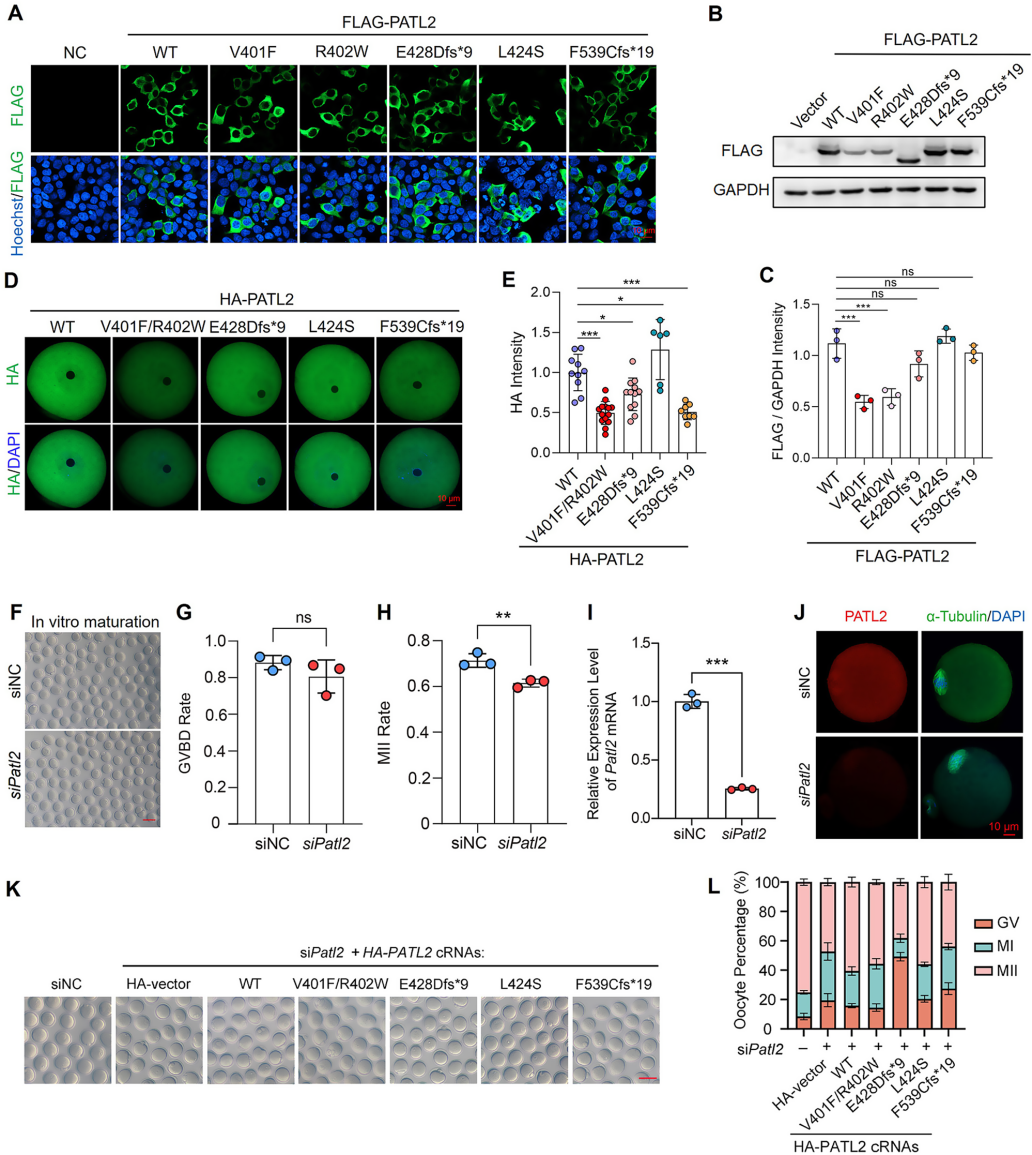
Effects of *PATL2* gene mutations on protein expression and localization in cells and oocytes. **(A)** Representative immunofluorescence images showing the expression and localization of FLAG-tagged PATL2 protein (green) in 293T cells. The cells were transfected with vectors expressing wild-type (WT) or mutant PATL2 (V401F, R402W, E428Dfs*9, L424S, and F539Cfs*19). DAPI (blue) was stained for visualization of DNA. Scale bar = 10 μm. **(B)** Western blot showing the expression levels of FLAG-tagged PATL2 in 293T cells transfected with vectors expressing WT or mutant PATL2 proteins. The blot was probed with anti-FLAG and anti-GAPDH antibodies, with GAPDH serving as a loading control. **(C)** Bar graph quantifying the intensity of FLAG-tagged PATL2 protein relative to GAPDH from the western blot analysis in **(B)**). Data are expressed as mean ± SD. Two-tailed Student’s *t*-test; ***P < 0.001, *P < 0.05, ns: not significant. **(D)** Representative immunofluorescence images showing the expression and localization of HA-tagged PATL2 protein in oocytes from mice. Fully-grown oocytes (GV stage) were microinjected with cRNAs encoding WT or mutant PATL2 (V401F/R402W, E428Dfs*9, L424S, F539Cfs*19). HA (green) and DAPI (blue) staining indicate the localization of PATL2 and the nuclei, respectively. Scale bar = 10 μm. **(E)** Bar graph quantifying the intensity of HA-tagged PATL2 protein in oocytes from **(D)**. Data are expressed as mean ± SD. Two-tailed Student’s *t*-test; ***P < 0.001, *P < 0.05. (**F**) The bright-field images showing the oocyte stages after 16 h of in vitro maturation in siNC or siPatl2 group. Scale bar = 100 μm. (**G-H**) The bar graphs showing the GVBD rate **(G)** and MII rate **(H)** after siNC or siPatl2 microinjection in GV oocytes. Three independent experiments were performed. Data are expressed as mean ± SD. Two-tailed Student’s t-test; **P < 0.01; ns, no significance. **(I)** RT-qPCR showing the *Patl2* mRNA level in MII oocytes with siNC or *siPatl2* siRNA microinjection. Data are expressed as mean ± SD. Statistical significance was determined using unpaired *t*-test; ***P < 0.001. **(J)** Immunofluorescence images showing the subcellular localization of PATL2 (red) and FITC-α-Tubulin (green) in MII oocytes undergoing knockdown of *Patl2* from GV stage. DAPI was used to stain chromosome (blue). Scale bar = 10 μm. **(K)** The bright-field images showing the oocytes after microinjection of negative control or mouse *Patl2* siRNAs combined with or without wild-type human HA-PATL2 or HA-PATL2 variants cRNAs and culture in medium with 2.5 µM milrinone for 12 h, followed by release to maturation (*n* > 120 oocytes each group). Scale bar = 100 μm. **(L)** The bar graphs showing the percentage of GV, MI and MII in K. Data are expressed as mean ± SD

Next, we used HEK293T cells to determine the functional properties of these *PATL2* variants compared to wild-type *PATL2*. We observed that the three *PATL2* variants had the similar subcellular localization with wild-type PATL2 in the cytoplasm by immunofluorescence (Fig. 3A). However, these mutations led to decreased PATL2 protein levels, especially those of the p.V401F, p.R402W, and p.E428Dfs*9 variants (Fig. 3B-C). We also microinjected cRNAs of HA-PATL2 or different variants into mouse GV oocytes, the localization was not changed but the protein of PATL2 variants were significantly decreased (Fig. 3D-E).

Next, we examined the functional impact of *PATL2* mutations on oocyte meiosis. We firstly microinjected siRNAs targeting negative control (siNC) or mouse *Patl2* (si*Patl2*) using mouse fully-grown GV oocytes. In si*Patl2* group, the GV breakdown was unaffected; however, the percentage of MII oocytes was slightly decreased compared to the siNC group (Fig. 3F-H). RT-PCR and immunofluorescence analysis confirmed efficient depletion of *Patl2* following siRNA treatment (Fig. 3I-J). We then microinjected the *HA-PATL2* cRNAs and various mutant cRNAs to assess their ability to rescue oocyte maturation. As expected, wild-type HA-PATL2 successfully rescued the MII rate, whereas the mutant variants exhibited reduced rescuing effects (Fig. 3K-L). Notably, two frameshift variants, HA-PATL2^E428Dfs*9^ and HA-PATL2^F539Cfs*19^, completely failed to rescue oocyte maturation compared to *siPatl2* group (Fig. 3K-L). These findings indicate that PATL2 mutations primarily result in protein instability and a consequent functional loss in varying degrees.

### Impact of *PATL2* variants on the protein interactome and cell cycle regulation

**Fig. 4:**
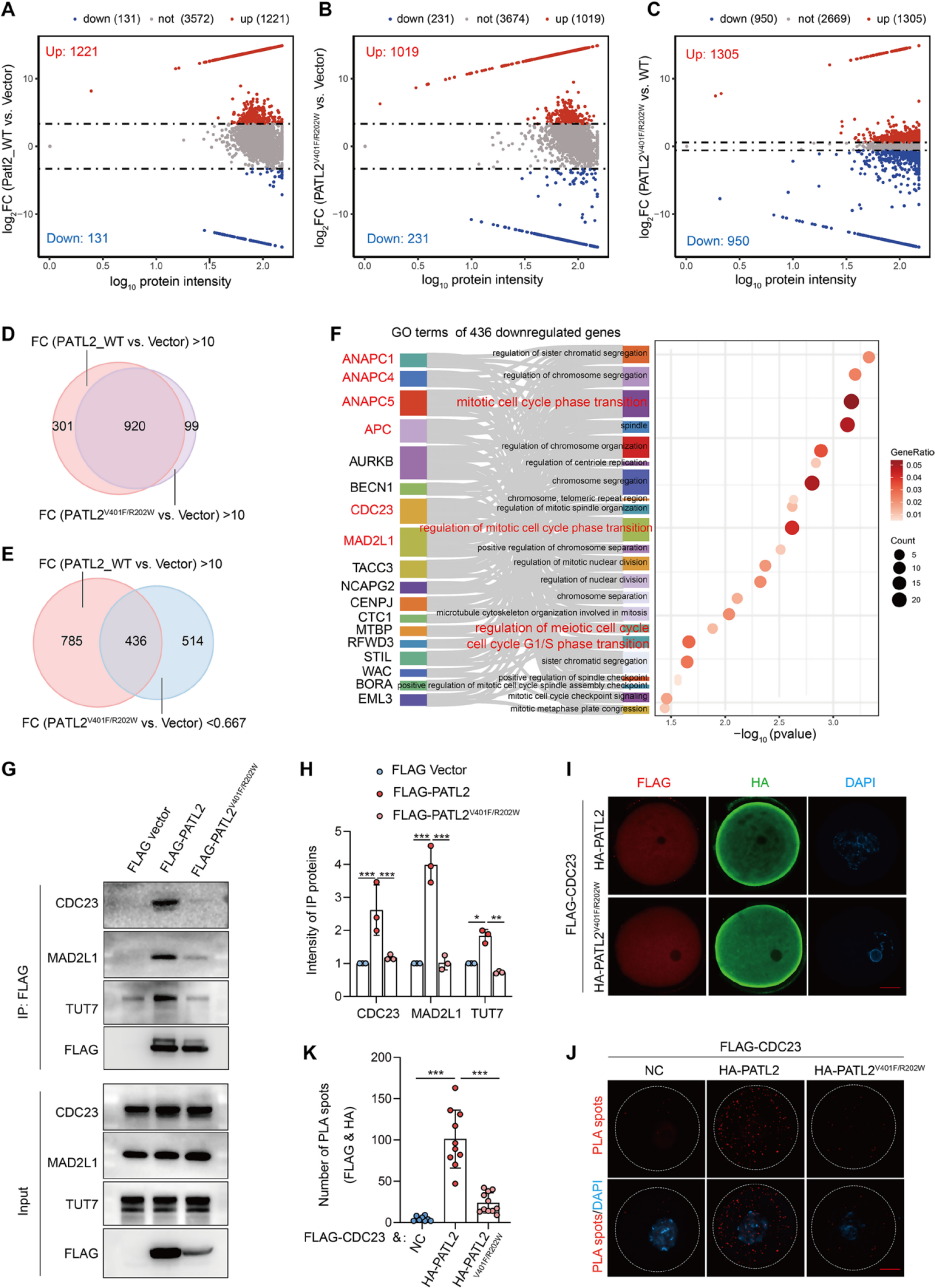
The altered protein interactome caused by PATL2 mutations associated with oocyte meiotic maturation **(A-C)** Scatter plots illustrating differential protein interactions in cells expressing FLAG-tagged wild type (WT) PATL2 relative to vector control (Vec) in **(A)**, PATL2^V401F/R402W^ mutant (Mut) relative to Vec **(B)**, and PATL2^V401F/R402W^ mutant relative to WT PATL2 **(C)**. The upregulated (red) and downregulated (green) interacting protein numbers were indicated. Ten-fold was set as a threshold in **(A)** and **(B)**, and three-fold was used as a threshold in **(C)**. **(D)** The Venn diagram illustrating the number of shared and unique interacting proteins in WT vs. Vec and Mut vs. Vec comparisons exhibiting a fold change greater than 10 in wild-type versus vector **(A)** and mutant **(B)** conditions. **(E)** Venn diagram showing the overlap of proteins interacting with WT PATL2 but reduced binding intensity in mutant PATL2. **(F)** Bubble chart displaying biological process terms for downregulated proteins in PATL2 mutant conditions. **(G)** Representative Western blot images showing the interaction between FLAG-PATL2 (both WT and mutant) and CDC23, MAD2L1 and TUT7. **(H)** Quantitative analysis of coimmunoprecipitation intensity for CDC23, MAD2L1 and TUT7, compared across FLAG vector, FLAG-PATL2, and mutant conditions, illustrating altered affinity in mutant samples. **(I)** Representative immunofluorescence images showing the subcellular localization of FLAG-CDC23 (red) co-expressed with HA-PATL2 or PATL2^V401F/R402W^ (green) mutant in GV oocytes. DAPI (blue) indicates the chromosome. Scale bar = 10 μm. **(J-K)** Immunofluorescence images and quantitative analysis of proximity ligation assay (PLA) signals in GV oocytes co-expressing FLAG-CDC23 with either HA-tagged wild-type PATL2 or mutant PATL2^V401F/R402W^ mutant, indicating decreased interaction between HA-PATL2 ^V401F/R402W^ mutant and FLAG-CDC23 in oocytes. Scale bar = 10 μm

Since the typical OMD observed in the patient from family 1 harboring two *PATL2* variants, we constructed an HA-tagged PATL2^V401F/R402W^ plasmid to assess differences in protein interactions compared to the wild-type PATL2. Using co-immunoprecipitation followed by mass spectrometry (MS/MS), we identified 1221 proteins that exhibited a ten-fold greater interaction intensity with the wild-type PATL2 relative to the vector control (Fig. 4A). This analysis revealed a total of 1019 PATL2^V401F/R402W^-binding proteins with at least a ten-fold increased interaction intensity relative to the HA vector group (Fig. 4B). Further comparison between the HA-PATL2^V401F/R402W^ mutant and the HA-tagged wild-type PATL2 identified 1305 up-regulated and 950 down-regulated binding proteins (Fig. 4C). Approximately 920 proteins had protein interactions between wild-type or PATL2^V401F/R402W^ mutant (Fig. 4D). Notably, 436 proteins demonstrated decreased interaction intensity in the PATL2^V401F/R402W^ mutant compared to the wild-type PATL2 with at least a 30% reduction in binding intensity (Fig. 4E). These 436 proteins were subjected to gene ontology (GO) analysis, the PATL2^V401F/R402W^ mutation-affected proteins are primarily involved in critical biological processes, such as the cell cycle, regulation of sister chromatid segregation, and regulation of meiotic cell cycle, exemplified by CDC23, MAD2L1, and APC1 (Fig. 4F).

Co-immunoprecipitation experiments demonstrated that ectopic PATL2 interacts robustly with endogenous proteins such as CDC23 and MAD2L1 in 293T cells (Fig. 4G-H). However, the binding level of the PATL2^V401F/R402W^ mutant with these proteins was significantly reduced (Fig. 4G-H). We further verified the interaction in oocyte through microinjecting cRNAs mix of FLAG-CDC23 and HA-PATL2 or HA-PALT2^V401F/R402W^ mutant (Fig. 4I). FLAG-CDC23 and HA-PATL2 (both WT and PALT2^V401F/R402W^ mutant) displayed uniform distribution in both nucleus and cytoplasm (Fig. 4I). Consistently, proximity ligation assays (PLA) showed that significant PLA spots were observed in wild-type HA-PATL2 and FLAG-CDC23, however, the PLA spots number was significantly decreased between HA-PATL2^V401F/R402W^ and FLAG-CDC23 (Fig. 4J-K). These results indicate that PATL2 ^V401F/R402W^ variant affects the protein interaction with cell cycle related proteins.

### PATL2 binds with CDC23 and stabilizes the CDC23 protein level in cells and mouse oocytes

**Fig. 5:**
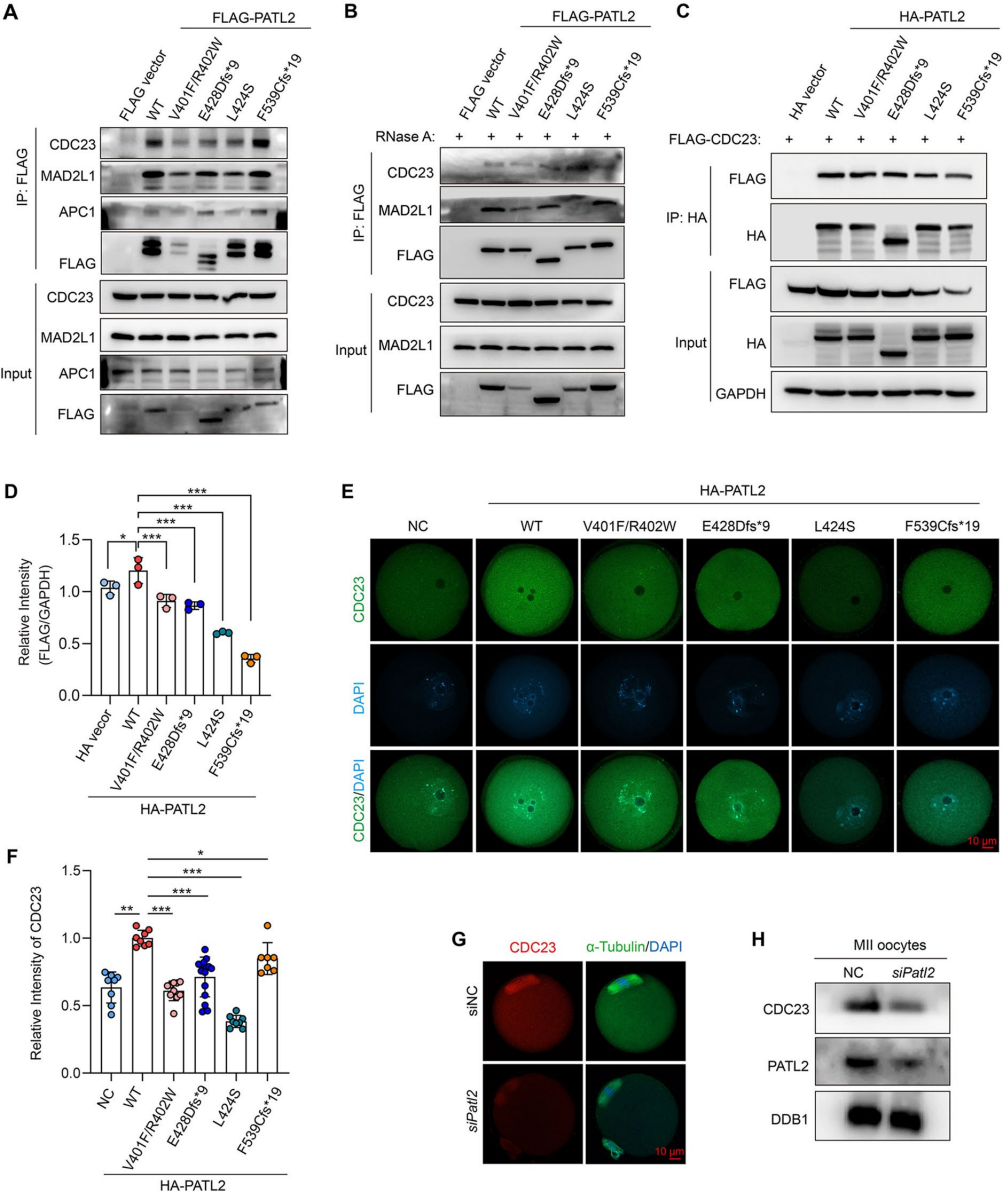
PATL2 interacts with CDC23 and stabilizes its protein level. **(A-B)** Western blot analysis showing the co-immunoprecipitation (coIP) results of CDC23, MAD2L1, and APC1 using an anti-FLAG antibody in cells expressing FLAG-tagged wild-type (WT) or various mutant forms of PATL2 without RNase A **(A)** or with 50 ng/ul RNase A treatment **(B)**. **(C)** Western blot results following CoIP assay showing the interaction of PATL2 and CDC23 in cells expressing HA-tagged PATL2 variants with FLAG-CDC23. **(D)** Quantification of FLAG-CDC23 intensities normalized to GAPDH in input in **(B)**. Statistical relevance is indicated using one-way ANOVA; *P < 0.05; ***P < 0.001. **(E)** Immunofluorescence staining of CDC23 (green) in GV oocytes (*n* > 30) overexpressing HA-tagged WT and mutant *PATL2*. DAPI was used to stain nuclei (blue). Scale bar = 10 μm. **(F)** Quantification of fluorescence intensity of CDC23 from **(D)**. Data are expressed as mean ± SD. Statistical significance was determined using a one-way ANOVA; *P < 0.05, **P < 0.01, ***P < 0.001. **(G)** Immunofluorescence images showing the subcellular localization of and CDC23 (red) and FITC-α-Tubulin (green) in MII oocytes undergoing knockdown of *Patl2* from GV stage. DAPI was used to stain chromosome (blue). Scale bar = 10 μm. **(H)** Western blot analysis of PATL2 and CDC23 on MII oocytes with or without *Patl2* knockdown. DDB1 is used as a loading control

Using co-immunoprecipitation, we found that ectopic FLAG-PATL2 could interact with endogenous CDC23, MAD2L1 and APC1, whereas it was decreased in *PATL2* mutant groups in different extent (Fig. 5A). The interaction between PATL2 and CDC23 or MAD2L1 were not affected with RNase A treatment in co-immunoprecipitation assay (Fig. 5B), indicating their interactions independent of RNAs. Since CDC23 is a key protein for oocyte cell cycle progression and the biallelic mutations in CDC23 cause human OMD and female infertility [17], we determined whether CDC23 is a downstream effector of PATL2 in cell cycle regulation. Ectopic HA-PATL2 and FLAG-CDC23 in 293T cells interacted with each other, and the binding intensity displayed slightly alteration in mutant groups (Fig. 5C). Notably, wild-type PATL2 overexpression increased the protein level of ectopic FLAG-CDC23 protein level, whereas this effect was mild in PATL2-mutant groups (Fig. 5C-D).

Next, we tested whether PATL2 stabilizes CDC23 protein level through protein interaction. We performed CHX chasing assay, and found that PATL2 could stabilize the protein degradation of CDC23, whereas the effect of PATL2 mutants on CDC23 degradation is significantly impaired (Figure S2A-B). Next, we microinjected cRNAs of HA-PATL2 and five variants into mouse GV oocytes and determined the endogenous CDC23 level. We found that PATL2 overexpression in GV oocytes increased the endogenous CDC23 protein level, whereas CDC23 levels were significantly decreased in *PATL2* mutant groups (Fig. 5E-F). We also examined the localization and expression of CDC23 in *Patl2*-knockdown (*siPatl2*) oocytes. *Patl2* knockdown did not change the localization of CDC23 in cytoplasm and at the spindle (Fig. 5G). However, the fluorescent signal of CDC23 was remarkably reduced (Fig. 5G), and immunoblotting confirmed that the protein level of CDC23 was significantly decreased in oocytes with *siPatl2* microinjection (Fig. 5H). Taken together the results of PLA in oocytes, these results indicated that PATL2 binds with and stabilizes the CDC23 protein level in oocytes.

### PATL2 maintains the protein levels of APC1 and MAD2L1 in mouse oocytes

We also examined the protein levels of other two interacting proteins (APC1 and MAD2L1) of CDC23. As expected, the protein levels of APC1 and MAD2L1 were significantly reduced in fully-grown GV oocytes following *Patl2* knockdown (Figure S2C). Immunofluorescence analysis further confirmed that *Patl2* knockdown led to a decrease in protein intensity of both MAD2L1 and APC1 (Figure S2D-G). These results indicate that PATL2 is crucial for the maintenance of APC1 and MAD2L1 protein levels in oocytes.

### Human PATL2 governs maternal mRNA homeostasis during human oocyte maturation

**Fig. 6:**
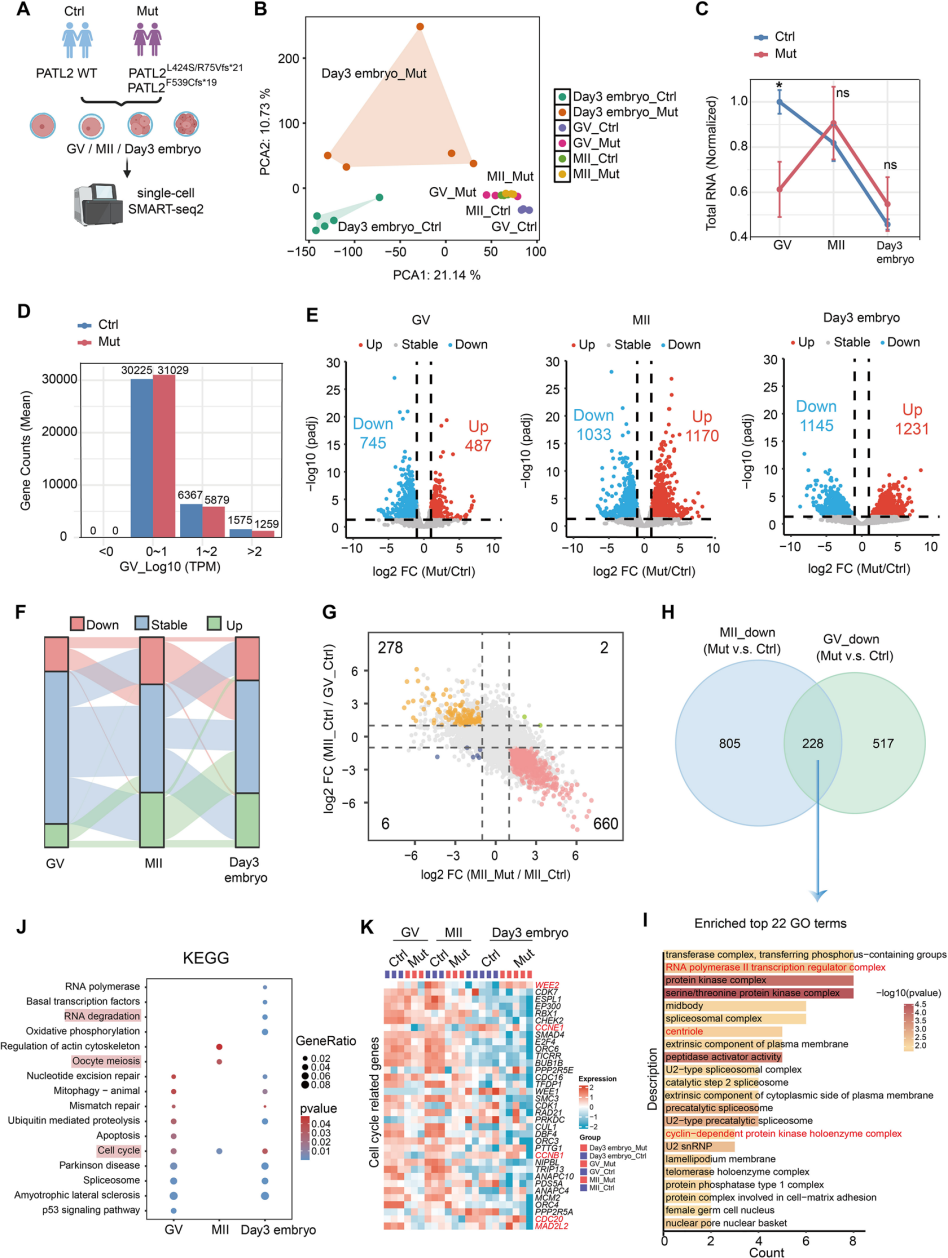
Transcriptomic profiling of oocytes and early-stage embryos harboring *PATL2* mutations. **(A)** Experimental design diagram depicting the groups studied: control (Ctrl) and *PATL2* mutant (Mut), including the specific mutations PATL2^L424S/R75Vfs*21^ and PATL2^F253Cfs*19^. Samples were collected for RNA-seq including germinal vesicle (GV) oocytes, metaphase II (MII) oocytes, and day 3 embryos. **(B)** Principal component analysis (PCA) illustrating transcriptomic segregation between control and *PATL2*-mutant samples, labeled by developmental stage. **(C)** Graphical representation of normalized total RNA reads using TPM across developmental stages between control and PATL2-mutant samples. Two-tailed Student’s *t*-test; *P < 0.05, ns: no significance. **(D)** Bar graph showing the mean transcript counts based on the gene expression level. **(E)** Volcano plots detailing differential expression genes across the GV, MII, and day 3 embryo stages between control and PATL2-mutant samples. The upregulated (red) and downregulated (blue) gene numbers were indicated. **(F)** Sankey diagram representing changes in expression status across the GV, MII, and day 3 embryo stages between control and PATL2-mutant samples, highlighting the continuity and shift between expression categories. **(G)** Quadrant diagram delineating the shared gene counts between MII/GV stages and PATL2 mutants relative to controls using log2 fold change in TPM values over 1. **(H)** Venn diagram showing the distribution and overlap of downregulated transcripts between MII and GV stages under PATL2 mutant conditions, as compared to controls. **(I)** Bar chart showing enriched Gene Ontology (GO) terms of the downregulated genes both in GV and MII oocytes with PATL2 mutation. **(J)** KEGG pathways enrichment of down-regulated genes at various developmental stages. **(K)** Heatmaps showing expression patterns of cell cycle related genes across the GV, MII, and day 3 embryo stages between control and PATL2-mutant samples, colored by relative expression levels from low (blue) to high (red)

Previous study has reported that *Patl2* loss affects RNA homeostasis in mouse oocyte [31]. Our co-IP and MS/MS results showed that PATL2 interacts with TUT7 and TUT4 (Fig. 4G and Table S4), two terminal uridylyltransferases with redundancy in regulation of RNA degradation. To determine whether PATL2 also regulates mRNA homeostasis in human oocytes, we performed low-input RNA-seq using GV, MII oocytes and day 3 early embryos from unidentified control patients (referred as WT group in Fig. 6). These samples include one GV, one MII, and five arrested embryos from the patient in family 3 with PATL2^L424S/R75Vfs*21^, and two GV and two MII oocytes with large polar body from the patient from family 5 with PATL2^F539Cfs*19^ (Fig. 6A and Figure S3A). Using PCA analysis, the transcriptome of *PATL2* mutant GV oocytes or day 3 early embryos were significantly different from those of control (Ctrl) patients (Fig. 6B), whereas the distribution of MII oocytes between control and *PATL2*-mutant groups were comparable (Fig. 6B). The mutation of *PATL2* hardly affects the *PATL2*’s mRNA level in GV oocytes but it was upregulated in MII and early embryos at day 3 (Figure S3B). After calibration with ERCC spike-in, we firstly compared the total mRNA level using TPM (Transcripts Per Kilobase per Million mapped reads) between control and *PATL2* mutant samples. In control group, the total mRNA levels were gradually decreased from GV oocytes, MII oocytes to day 3 early embryos (Fig. 6C). In contrast, the mRNA level was significantly decreased in *PATL2*-mutant oocytes at GV stage (Fig. 6C). However, the total mRNA levels of MII oocytes or day 3 early embryos were comparable between control and PATL2 mutant groups (Fig. 6C). We analyzed the expression distribution of detected transcripts, and found that the gene counts with TPM over 10 in *PATL2*-mutant GV oocytes is much less than those in control group (Fig. 6D), which indicate that the less mRNA dosage in *PATL2*-mutant GV oocytes is due to the low mRNA expression of highly-expressed genes. In contrast, in MII stage or day 3 embryos, the high-expression genes (TPM > 10) number in *PATL2*-mutant group were higher than control group (Figure S3C).

Detailed comparative analysis supported an abnormal transcriptome in *PATL2*-mutant GV oocytes, with significant differences (745 transcripts with reduced abundance, 487 with increased abundance) using a threshold with |log2fold-change| > 1 and padj < 0.05 (Fig. 6E-F). These differences became more pronounced in the MII oocytes (1033 transcripts with reduced abundance, 1170 with increased abundance) and day 3 embryos (1145 transcripts with reduced abundance, 1231 with increased abundance) (Fig. 6E-F). In addition, a quadrant diagram revealed that among 1170 transcripts significantly upregulated in *PATL2*-mutant MII oocytes, 660 genes should be dramatically degraded across oocyte maturation (Fig. 6G), indicating an obvious delay in mRNA clearance during GV-MII transition with *PATL2* mutations (Fig. 6G). We also analyzed the percentage of zygotic genome activation (ZGA) and M-decay genes in down-regulated genes in day 3 embryos. We found 41% (457 in 1145 genes) downregulated genes in *PATL2*-mutant day 3 embryos were ZGA genes, and 27% (336 in 1231 genes) upregulated genes were M-decay genes (Figure S3D). These results suggested that PATL2 is critical for regulating the mRNA dosage in human immature oocytes and the mRNA degradation in MII oocytes and early embryos.

We also analyzed the conserved down-regulated genes (228 genes) both in GV and MII oocytes (Fig. 6H), which may partially explain the GV arrest. The gene ontology (GO) analysis found that the transcripts relative to RNA polymerase II transcription regulator complex, protein kinase complex and cyclin-dependent protein kinase holoenzyme complex were affected (Fig. 6I). Analysis of functional enrichment data of down-regulated genes with *PATL2* mutations at each stage showed that a variety of fundamental biological processes are involved: RNA splicing, mRNA processing and mitochondrial translation at the GV stage (Figure S3E); mRNA processing, chromosome segregation, actin cytoskeleton and spindle organization at the MII stage (Figure S3F); ncRNA processing and ribosome biogenesis in day 3 embryos (Figure S3G). Notably, pathways associated with cell cycle were significantly enriched in down-regulated genes of *PATL2*-mutant group at the GV, MII stage and day 3 embryos (Fig. 6J-K), and RNA polymerase and RNA degradation was only enriched in day 3 embryos (Fig. 6J). We analyzed the expression level of PATL2-binding proteins relative to oocyte meiosis using the TPM in RNA-seq. All the gene expression (*CDC23*, *ANAPC1*, *ANAPC2*, *ANAPC5* and *MAD2L1*) were comparable between two group at each stage (Figure S3H). However, we noted that the mRNA levels of *CCNB1* and *CCNE1* were significantly decreased in GV oocytes (Fig. 6K and Figure S3I), and *CDC20* was decreased in MII oocytes with *PATL2* mutation (Fig. 6K and Figure S3I). *WEE2*, a maternal gene with mRNA degradation from MII to day 3 embryo, was upregulated in *PATL2*-mutatnt embryos at day 3 (Fig. 6K and Figure S3I). In summary, these results suggested that *PATL2* mutations affected cell cycle-related genes expression and degradation required for human oocyte maturation and early embryonic development.

### *PATL2* mutation impeded mRNA decay in human oocytes and embryos

**Fig. 7:**
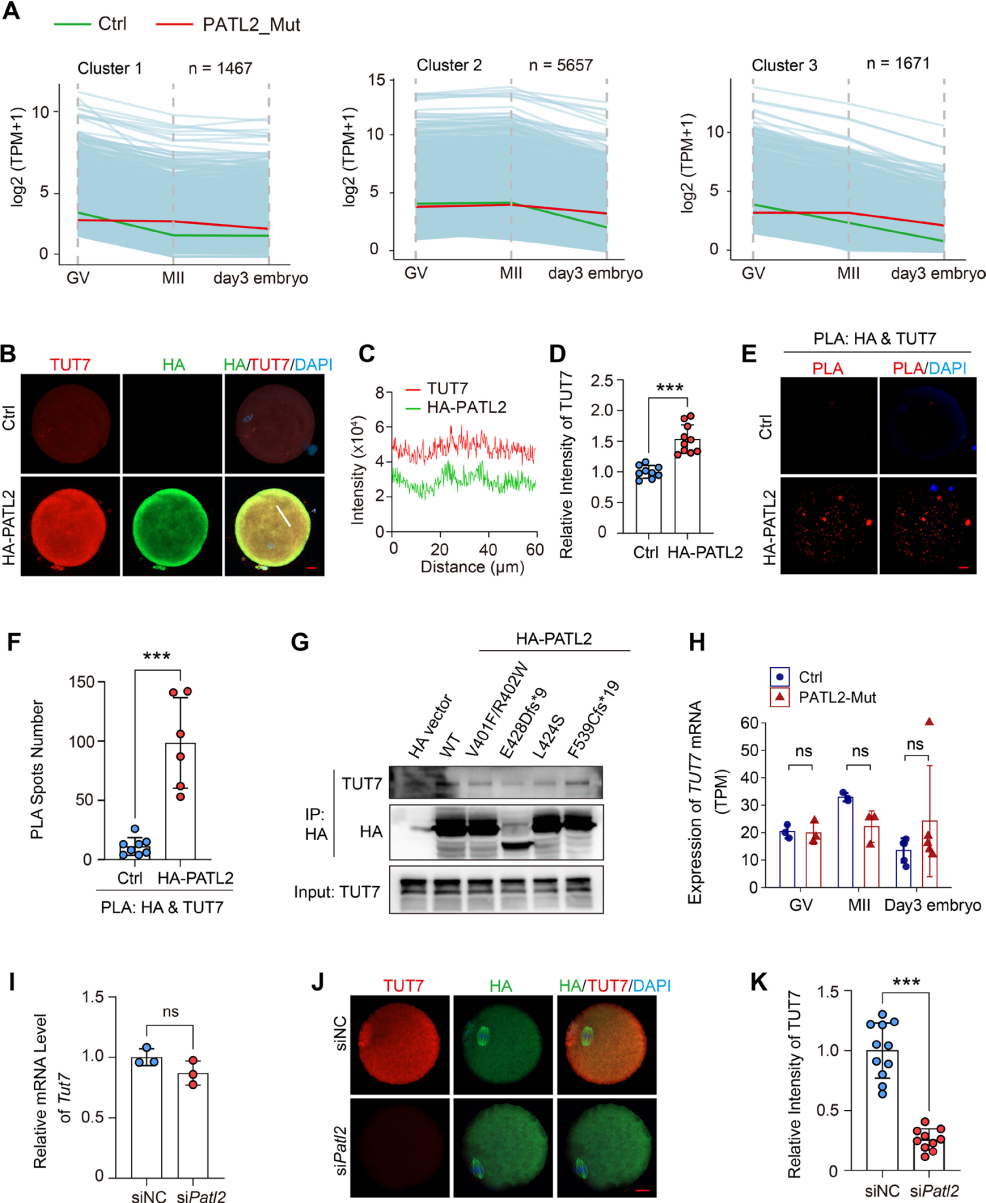
PATL2 binds with TUT7 in oocytes and promotes maternal mRNA decay. **(A)** Degradation patterns of human maternal transcripts at the GV, MII oocytes and day 3 embryos. The green line and red line represent the median expression levels of the cluster in control and PATL2-mutant group, respectively. **(B)** Representative immunofluorescence images showing the colocalization of ectopic HA-PATL2 (green) and TUT7 (red) in human MII oocytes. The human MII oocytes with or without microinjection with *HA-PATL2* cRNAs were stained with anti-HA and anti-TUT7 antibody co-stained with DAPI. Scale bar = 20 μm. **(C)** Fluorescence intensity of HA-PATL2 and TUT7 of the white line in **(B)** was measured. **(D)** The graph showing the intensity of TUT7 in **(B)**. **(E)** In situ proximity ligation assays (PLA) showing the interaction between HA-PATL2 and TUT7 in human oocytes with or without microinjection of HA-PATL2 cRNAs. Scale bar = 20 μm. **(F)** The graph showing the PLA spots numbers quantified by Image J software. **(G)** The western blot results showing the coimmunoprecipitation of ectopic expression of HA-PATL2 and PATL2 variants in 293T cells. The anti-HA and anti-TUT7 were immunoblotted. **(H)** Bar graph showing expression levels of TUT7 in RNA-seq of PATL2-mutant and control GV, MII, and Day 3 embryos. **(I)** RT-qPCR results showing the *Tut7* mRNA level in MII oocytes with siNC and si*Patl2* knockdown (*n* = 3 biological replicates). **(J)** Representative images showing TUT7 (red) in MII oocytes microinjected with siNC and si*Patl2* at GV oocyte. The spindles were stained with FITC-α-Tubulin (green) and chromosomes were stained with DAPI (DAPI). Scale bar = 20 μm. **(K)** Quantifications of TUT7 intensity in **(I)**. An average fluorescence intensity is measured by single oocyte and is plotted as a single dot. Data in **(D)**, **(F)**, **(H)**, **(I)** and **(K)** are expressed as mean ± SD. Two-tailed Student’s t-test; ***P < 0.001; ns, no significance

To investigate the impact of *PATL2* mutations on maternal mRNA degradation in human oocytes and embryos, we analyzed the mRNA dynamics in GV oocytes, MII oocytes, and day 3 embryos using our RNA-seq data. Maternal mRNAs with reliable sequence annotations and a TPM > 2 in the control group at any stage (a total of 38,167 genes) were selected for analysis. Genes were classified into nine categories based on changes in their mRNA levels, defined as a significant 2-fold or greater downregulation or upregulation between stages. Three clusters of degraded maternal mRNAs were identified: Cluster I (1,467 genes), which degraded from GV to MII and remained stable post-fertilization; Cluster II (5,657 genes), which remained stable from GV to MII but degraded in day 3 embryos; and Cluster III (1,671 genes), which exhibited continuous degradation from GV to day 3 embryos (Fig. 7A). In oocytes and embryos with *PATL2* mutations, mRNA decay was impaired across all three clusters, suggesting a defect in maternal mRNA degradation.

**Fig. 8:**
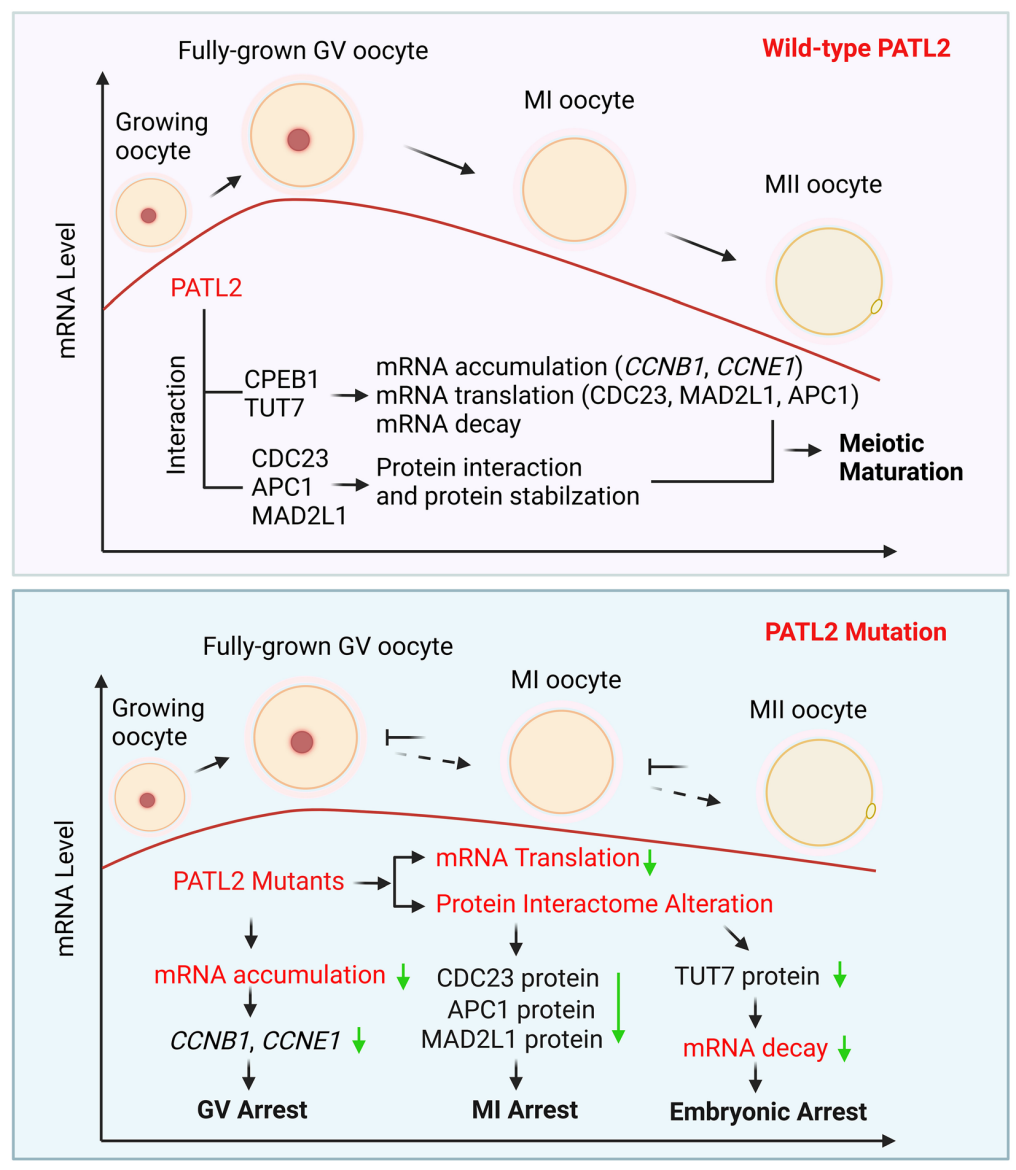
Proposed model showing *PATL2* mutations affect human oocyte maternal mRNA homeostasis and the protein interaction of cell cycle related proteins to cause oocyte meiosis defect and early embryonic arrest. PATL2 interacts with CPEB1 and TUT7 in human oocytes to maintain mRNA homeostasis, including mRNA accumulation, mRNA translation and degradation. *PATL2* mutations affected mRNA storage in human germinal vesicle (GV) oocytes and mRNA decay during maturation and in early embryos. *PATL2* mutations induce a reduction in *CCNB1* and *CCNE1* mRNA levels in GV oocytes, which may be linked to GV arrest. *PATL2* mutation alters the protein translation and interactome of PATL2, predominantly affecting cell cycle-related proteins, such as CDC23. PATL2’s interaction with and stabilization of CDC23 and TUT7 in oocytes elucidate the mechanisms behind the mutation-induced MI arrest and mRNA decay defect, respectively

The remaining clusters included: Cluster IV (2,105 genes), stable across all stages; Cluster V (231 genes), which elevated from GV to MII and remained stable from MII to day 3 embryos; Cluster VI (2,656 genes), stable from GV to MII but elevated from MII to day 3 embryos; Cluster VII (244 genes), consistently elevated from GV to the embryonic stage; Cluster VIII (1,540 genes), which degraded from GV to MII and elevated post-fertilization; and Cluster IX (892 genes), which elevated from GV to MII but degraded after fertilization (Figure S4A). Notably, the mRNAs transcribed during zygotic genome activation (ZGA), identified in Cluster VI, were impaired in day 3 embryos with maternal PATL2 mutations. These results demonstrate that *PATL2* mutations disrupt both mRNA decay and ZGA (Fig. 8).

### PATL2 interacts with CPEB1 and TUT7 in human oocytes and embryos

Given the evidence that PATL2’s interaction with CPEB1 to modulate RNA homeostasis in mouse oocytes [31] and interaction with TUT7 in Fig. 4G, we extended these findings to human oocytes and embryos. We investigated the roles of PATL2 in mRNA accumulation and decay by examining its interactions with CPEB1 and TUT7, respectively. Through extraction of the TPM of *CPEB1* mRNAs in our RNA-seq data (Table S5), we found *CPEB1* mRNAs are comparable between control and PATL2-mutatnt oocytes, but was upregulated in day 3 embryos (Figure S4B), indicating degradation defect. Following microinjection of HA-tagged PATL2 into human oocytes or 3PN zygotes, we observed colocalization of HA with both CPEB1 (Figure S4C). Proximity ligation assays (PLA) using antibodies against HA and CPEB1 revealed a higher number of PLA signals in the HA-PATL2 group compared to controls (Figure S4D-E). Similar to human oocytes, the mRNA level of *Cpeb1* in mouse MII oocytes with *Patl2* knockdown was unaffected (Figure S4F). These suggest a conserved role of PATL2 and CPEB1 in maintaining mRNA accumulation across mouse and human oocytes.

Similarly, colocalization of HA-PATL2 and endogenous TUT7 was observed in human oocytes (Fig. 7B-C). Notably, HA-PATL2 overexpression induced a protein increase of TUT7 (Fig. 7D). The enhanced PLA signals were noted between HA-PATL2 and TUT7 in oocytes relative untreated control oocytes (Fig. 7E-F). Using coimmunoprecipitation, the wild-type PATL2 binds with TUT7, and the binding intensities between PATL2 mutants (except to PATL2^F539Cfs*19^) and endogenous TUT7 were decreased (Fig. 7G). We then evaluated the effects of PATL2 depletion on the protein level of TUT7. In human *PATL2*-mutant oocytes or early embryos, the mRNA levels of *TUT7* were comparable with control group (Fig. 7H). Similar with human *PATL2* mutation, *Patl2* knockdown using siRNAs did not induce the downregulation of *Tut7* mRNAs in mouse oocytes (Fig. 7I). However, *Patl2* knockdown induces the TUT7 protein decrease in mouse oocyte (Fig. 7J-K). Collectively, PATL2 is required for TUT7 protein level maintenance, and PATL2 probably collaborates with TUT7 to involve mRNA decay in human oocyte and early embryos.

### PATL2 is required for global mRNA translation in oocytes

We observed that PATL2 overexpression resulted in higher protein levels of CDC23 and TUT7, while PATL2 knockdown led to a reduction in the protein levels of CDC23, APC1, MAD2L1, and TUT7 without affecting their mRNA levels. These findings suggest that, in addition to promoting protein stabilization through protein interactions, PATL2 may also maintain protein levels through regulation of mRNA translation. We examined the new protein synthesis ability by HPG (L-homopropargylglycine, an amino acid analog of methionine) incorporation. Consistent with the previous study [31], the HPG assay revealed that *Patl2* knockdown resulted in a global decrease in mRNA translation in mouse GV oocytes (Figure S5A-B). In contrast, overexpression of human *HA-PATL2* cRNAs in *siPatl2* oocytes reversed PATL2 protein level and mRNA translation defect (Figure S5A-B). To further assess the impact of PATL2 on mRNA translation, we microinjected exogenous *GFP* cRNAs into siNC and *siPatl2* mouse GV oocytes and determined the GFP intensity at different timepoints (0 h, 6 h, 12 h and 24 h). The GFP signal intensity was significantly reduced in *Patl2*-knockdown oocytes at different timepoints (Figure S5C-D). These results underscore the essential role of PATL2 in facilitating efficient mRNA translation (Fig. 8).

## Discussion

In this study, we identified six mutations in *PATL2* among five independent primary infertility cases, including one novel missense mutation (c.1201G > T), two novel frameshift mutations (c.1284delA, c.1613 + 2_1613 + 3insGT), one recurrent mutation (c.223 − 14_223-2delCCCTCCTGTTCCA) and two recently reported missense mutations (c.1204 C > T and c.1271T > C) [38]. These affected individuals had similar phenotypes but also showed a multiplicity. The typical clinical phenotypes of these patients with *PATL2* mutation were the immature oocytes at GV or MI stage. Notably, only a minority of oocytes from families 2 and 5 reached MII stage, with fertilization observed solely in mature oocytes from a patient in family 5, albeit with subsequent embryonic arrest at early developmental stages. The patient from family 2 (II-1), identified as homozygous for the reported mutation (c.223 − 14_223-2delCCCTCCTGTTCCA, p.Arg75Valfs21), predominantly extruded mature oocytes, aligning with previously documented phenotypes [39]. Conversely, the patient from family 1 (II-1) carrying compound heterozygous mutations (c.1201G > T and c.1204 C > T) displayed the typical OMD phenotype with oocytes arrested at GV, MI, or presenting abnormalities. Another notable case from family 5 (II-1) with a homozygous frameshift mutation (c.1613 + 2_1613 + 3insGT, p. F539Cfs*19) exhibited similar GV-stage arrest and oocyte degeneration. Given the phenotypic conservation observed with the missense mutations in family 1, we specifically investigated mutations c.1201G > T and c.1204 C > T in *PATL2* (encoding PATL2^V401F/R402W^ proteins) to elucidate their potential impact on the protein interactome. Our findings indicate that different *PATL2* mutations variably affect protein levels and binding affinity with cell cycle-related proteins, potentially explaining the phenotypic variability observed in OMD. This multiplicity induced by different mutations is consistent with the previous finding [39–41].

*PATL2* mutations are primarily implicated in arresting oocyte development at the GV stage, occasionally extending to MI arrest. Despite extensive research, the molecular mechanisms by which PATL2 mutations induce these specific arrests remain poorly understood. Notably, three *Patl2* mutant mouse lines of *Patl2* —comprising knockout (KO) models and point mutations—were established by two independent research groups [31, 32]. However, the OMD phenotype of two KO mouse lines was mild in the in vivo ovulated oocytes but displayed decreased MII rate in vitro maturation. The two *Patl2* KO mice line showed early embryonic arrest [31, 32], a considerably milder phenotype compared to that observed in *PATL2*-mutant females. Given the scarcity of human oocytes, elucidating the mechanistic underpinnings of PATL2 mutation-induced oocyte maturation defects (OMD) poses significant challenges. In our study, we employed single-oocyte and embryo mRNA sequencing on *PATL2*-mutant GV and MII oocytes. The results revealed a significant reduction in the mRNA expression of key cell cycle-related genes, including *CCNB1*, *CCNE1*, and *CDC20*, predominantly in PATL2-mutant GV oocytes.

The understanding of oocyte meiosis has primarily been derived from murine models. In murine models, various cyclins, such as cyclins A1, A2, B1, B2, B3, and cyclin O, are crucial for oocyte meiosis progression through their interaction with CDK1 [9]. Notably, Cyclin B1 (encoded by CCNB1) and Cyclin B2 are considered primary partners of CDK1 during oocyte maturation [42]. The *Ccnb1*-null oocytes display meiosis II arrest failure [43], whereas *Ccnb2*-null oocyte exhibit severe delay in meiotic resumption and progression [44]. A complete meiotic resumption defect is induced by the simultaneous deletion of *Ccnb1* and *Ccnb2* [43]. Conversely, overexpression of MOS, which promotes Cyclin B1 translation in GV oocytes, can ameliorate GV arrest induced by PDE inhibitors [10]. It has long been believed a key role of CCNB1 in human oocyte meiosis. A study comparing young and aged human MII oocyte displayed the mRNA decrease of *CCNB1* [45]. Despite a significant decrease in *CCNE1* mRNA, the specific role of this cyclin in mouse or human oocyte development remains unexplored. CDC20, another decreased mRNA in *PATL2*-mutant oocytes, plays a conserved role in regulating oocyte meiosis from mice to human [15]. Several studies showed that biallelic *CDC20* mutations lead to mainly OMD phenotype [24, 46, 47]. Our study suggested that the mRNA decrease of *CCNB1* and *CDC20* may be partially responsible for the OMD induced by *PATL2* mutation.

In our study, we identified the cell cycle related proteins as the PATL2-interacting proteins using proteomic analysis, such as CDC23, MAD2L1, ANAPC1 (also known as APC1), ANAPC2, ANAPC4, and ANAPC5 (Figure S6A). The interaction between PATL2 mutants (PATL2^V401F/R402W^, PATL2^E428Dfs*9^ and PATL2^L424S^) and endogenous CDC23, APC1 or MAD2L1 showed significant decrease of interactions in 293T cells. Despite that the ectopic expression of FLAG-CDC23 showed similar binding level with PATL2^V401F/R402W^ in 293T cells, their interaction significantly decreased in oocytes (Fig. 4J-K). We found that wild-type PATL2 overexpression induces CDC23 protein increase, whereas the effect of PATL2 mutations significantly rendered the increase of CDC23 protein (Fig. 5C-F). Because PATL2 is required for global mRNA translation (Figure S5A-B), we suggest that the CDC23 protein increase or maintenance may be the dual effects of mRNA translation promotion and protein stabilization by PATL2. CDC23, also known as APC8, play key roles in manipulating both human and mouse oocyte meiosis progression [18]. Given that *Patl2* knockdown led to decreased protein levels of multiple cell cycle-related proteins, we suggest that microinjection of CDC23 cRNAs alone would be insufficient to rescue the OMD phenotype induced by PATL2 depletion or mutation. CDC23 homozygous mutations induced a decrease in CDC23 protein level and the accumulation of securin and cyclin B1 in oocytes, and AZ3146, an inhibitor of spindle assembly checkpoint, was able to partially rescue the OMD phenotype [17]. Similarly, in *Patl2*-knockdown oocytes, AZ3146 treatment rescued the MII percentage, although the MII oocytes with large polar bodies increased significantly (Figure S6B-C). We propose that the OMD phenotype induced by PATL2 mutation is not only due to decreased CDC23 levels but also involves the combined effects of mRNA and protein reductions in various cell cycle-related genes, contributing to GV and MI arrest.

The RNA-seq data of *PATL2*-mutant oocytes suggested a critical role for PATL2 in promoting mRNA accumulation and mRNA decay during human oocyte maturation. The GV oocytes of control and three *PATL2*-mutant oocytes from two individuals displayed distinctly different transcriptome from PCA analysis results. After ERCC normalization, the total mRNA amounts were significantly decreased in *Patl2*-mutant GV oocytes, which is consistent with that using *Patl2*-null GV oocyte in mice [31]. The human PATL2 showed colocalization and interaction with CPEB1 (Figure S4C-E), indicating the conserved role of PATL2 as a mRNA translation regulator with CPEB1. Interestingly, the mRNA amounts of MII oocytes and day 3 embryos were comparable. This result is consistent with the RNA-seq results using *Patl2*-null mouse oocytes [31], in which comparable mRNA amounts are probably due to 80% mRNAs degradation from wild-type GV to MII oocytes and the mRNA decay defect with *Patl2*-mutated MII oocytes. Consistently, our RNA-seq results of MII oocytes that *PATL2* mutations led to upregulation of mRNAs that should be degraded in MII stage oocytes (Fig. 6G) and day 3 embryos (Figure S3D). This revealed the key role of PATL2 on mRNA decay. Since previous studies demonstrated *PATL2* mutation affected *MOS* mRNA translation [41], leading to large polar body in some patients. Our group previously demonstrated that MOS mutation caused mRNA decay defect in MII oocytes [25], large polar body extrusion and early embryonic arrest [33]. Notably, PATL2 binds with TUT7 and TUT4, whereas the PATL2^V401F/R402W^ variant showed decreased binding intensity as shown in Fig. 4G and Table S4. TUT7 and TUT4 are two redundant uridylation enzymes to add mRNA 3’ uridylation [48]. Uridylation on the short poly (A) mRNAs mediated TUT7 and TUT4 is essential for maternal mRNA degradation during oocyte maturation [49] and mRNA clearance during early embryo development [50–52]. Our results also found that PATL2 maintains high protein level of TUT7 possibly through mRNA translation and protein stabilization. The protein interaction between PATL2 and TUT7 indicate that there is possibility of PATL2 involving the normal mRNA degradation processes directly during oocytes maturation. It requires further investigation about the detailed role of PATL2 on mRNA decay.

In summary, we identified three novel mutations in *PATL2* gene in infertile patients exhibiting OMD, expanding the genotype spectrum of OMD. Our research provides a global view of the PATL2 protein interactome and revealed PATL2 mutations affected the protein interactome, especially alteration with cell cycle proteins. Furthermore, we validate that PATL2 directly binds with and stabilizes CDC23 in oocyte to prevent MI arrest. We also provide first direct evidence in human oocytes using RNA-seq that *PATL2* variants influence mRNA accumulation in immature GV oocytes and impedes mRNA decay in human oocyte maturation and early embryonic development. These effects are likely mediated through the protein interactions between PATL2, CPEB1, and TUT7 (Fig. 8). This study highlights the critical role of PATL2 in regulating oocyte meiosis through maintaining mRNA homeostasis (including mRNA accumulation and translation) and protein stabilization of cell cycle related proteins. Our finding in this study will advance our understanding of OMD induced by PATL2 mutations.

## Conclusions

This study expands the spectrum of *PATL2* variants and provides pathogenic evidence for genetic counseling for female infertility. We demonstrate that PATL2 is essential for mRNA accumulation and mRNA decay in human oocytes, potentially through collaborating with CPEB1 and TUT7, respectively. Mutations in PATL2 lead to oocyte meiosis defects by directly binding to and stabilizing CDC23, and indirectly affecting the accumulation and translation of mRNAs related to cell cycle regulation, including *CCNB1*,* CDC23*,* APC1* and *MAD2L1*. This study provides new insights into the molecular mechanisms of OMD caused by PATL2 deficiency.

## Electronic supplementary material

Below is the link to the electronic supplementary material.

Supplementary Material 1

Supplementary Material 2

Supplementary Material 3

Supplementary Material 4

## Data Availability

There are six supplemental figures (Figure S1-S6) and five supplemental table (Table. S1-S5) in the present study. A summary of MS/MS and RNA-seq data generated in this study is shown in Table S4 and Table S5, respectively. The raw RNA-seq data have been deposited in the Genome Sequence Archive in National Genomics Data Center, China National Center for Bioinformation / Beijing Institute of Genomics, Chinese Academy of Sciences (GSA-Human: HRA008646).

## Abbreviations

OMD: Oocyte maturation defect
ART: Assisted reproductive technology
GV: Germinal vesicle
MI: Metaphase I
MII: Metaphase II
MPF: Maturation promoting factor
APC/C: Anaphase-promoting complex/cyclosome
ZGA: Zygotic genome activation
TPM: Transcripts Per Kilobase per Million mapped reads
