# Supplementary material for: PATL2 mutations affect human oocyte maternal mRNA homeostasis and protein interactions in cell cycle regulation
Source: Cell Biosci. 2024 Dec 31;14:157. doi: 10.1186/s13578-024-01341-2 (PMC11686847; doi:10.1186/s13578-024-01341-2)
Supplement: Supplementary file 3 — Supplementary Material 3 [file 13578_2024_1341_MOESM3_ESM.pdf]

Supplementary Figures

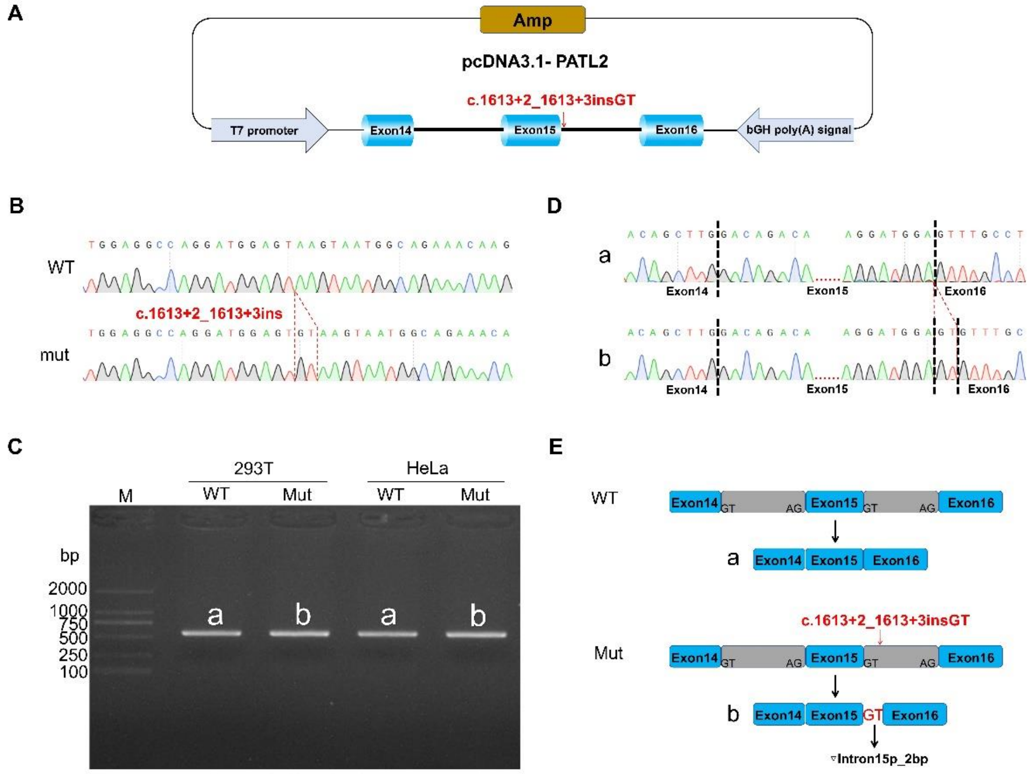

Figure S1. Experimental Validation of PATL2 Splice Site Mutation c.1613+2_1613+3insGT. **(A)** Diagram illustrating the splicing constructs used for validating PATL2 splice site mutations. The constructs include exons 14-16 with either wild-type or mutant sequences (c.1613+2_1613+3insGT). The T7 promoter and bGH poly(A) signal are shown, indicating the transcriptional and polyadenylation elements. **(B)** Sanger sequencing chromatograms of RNA extracted from cells transfected with wild-type (WT) or mutant (Mut) constructs. The red arrows highlight the insertion mutation c.1613+2_1613+3insGT, which disrupts normal splicing at the junction of exons 14 and 15. **(C)** Gel electrophoresis image of RT-PCR products from cells transfected with WT or mutant constructs. Lanes a and b correspond to WT and mutant constructs, respectively. The gel shows the expected sizes of the PCR products, indicating differences in splicing patterns between WT and mutant. **(D)** Diagram depicting the expected splicing outcomes for WT and mutant constructs. The wild-type sequence produces a correctly spliced transcript with exons 14, 15, and 16. In contrast, the mutant sequence with the c.1613+2_1613+3insGT insertion results in aberrant splicing, leading to the inclusion of a GT dinucleotide between exons 15 and 16.

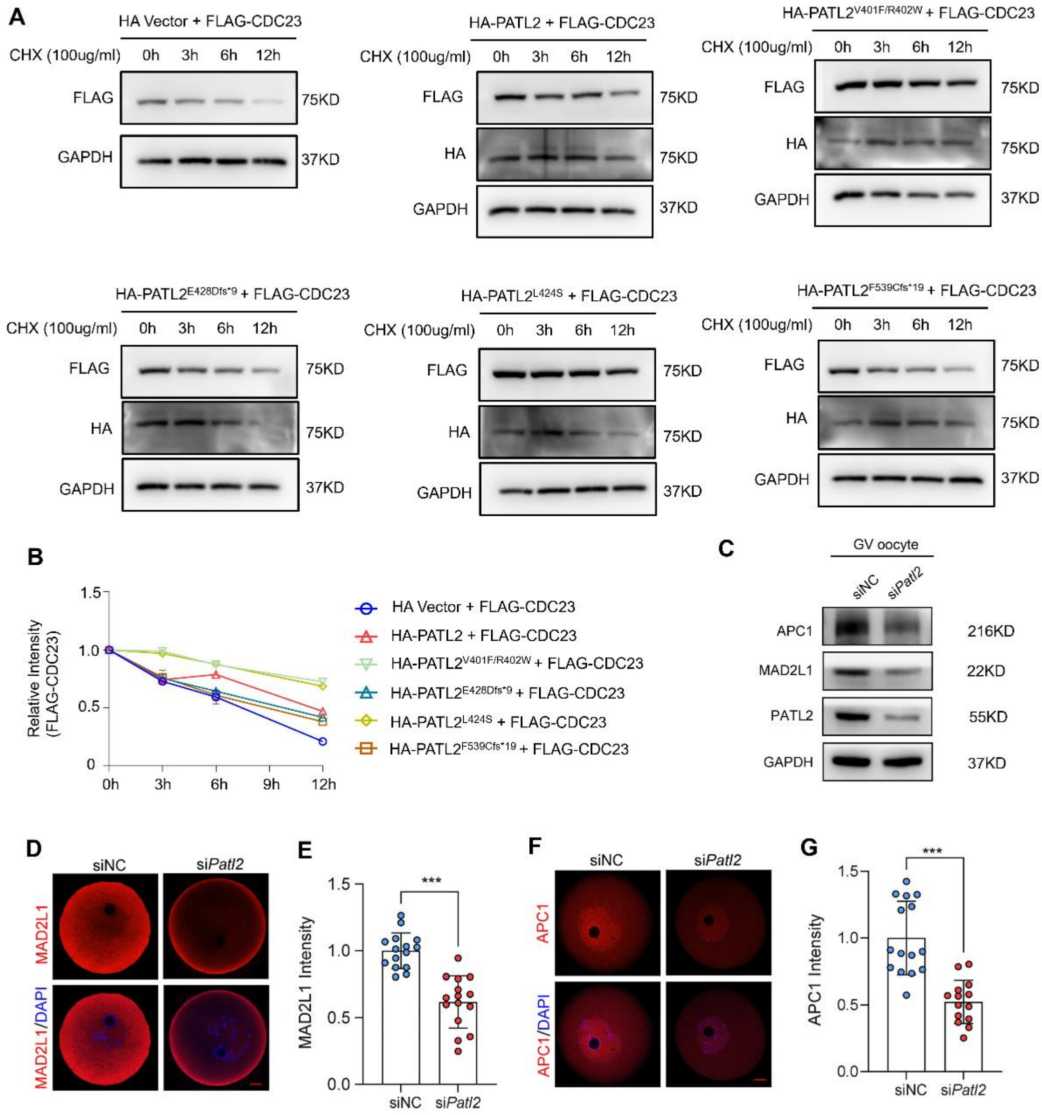

Figure S2. Effect of different *PATL2* variants on the stability of FLAG-tagged CDC23 protein and the effect of *Patl2* knockdown on MAD2L1 and APC1 protein level.

(A) Western blot analysis showing the degradation profiles of FLAG-CDC23 in cells transfected with either HA-tagged vector alone or various HA-tagged PATL2 mutants: L2424S, L2428Nfs*3, V401F/R402W, and F539Cfs*19. Cells were treated with cycloheximide (CHX, 100 μg/ml) to halt protein synthesis, and protein levels were assessed at 0, 3, 6, and 12 hours post-treatment. **(B)** Quantification of the relative intensity of FLAG-CDC23 bands normalized to GAPDH, plotted over the treatment duration. This graph illustrates the differential impact of each PATL2 mutation on the stability of FLAG-CDC23. **(C)** Western blot analysis of APC1, MAD2L1 and PATL2 in GV oocytes with or without *Patl2* knockdown. GAPDH is used as a loading control. **(D, F)** Immunofluorescence images showing the subcellular localization of MAD2L1 (red, D) and APC1 (red, F) in GV oocytes undergoing knockdown of *Patl2*. DAPI was used to stain nucleus (blue). Scale bar = 10 μm. **(E, G)** Quantifications of MAD2L1 intensity in (D) and APC1 intensity in (F). An average fluorescence intensity is measured by single oocyte and is plotted as a single dot. Data are expressed as mean ± SD. Two-tailed Student's t-test; ****P*<0.001.

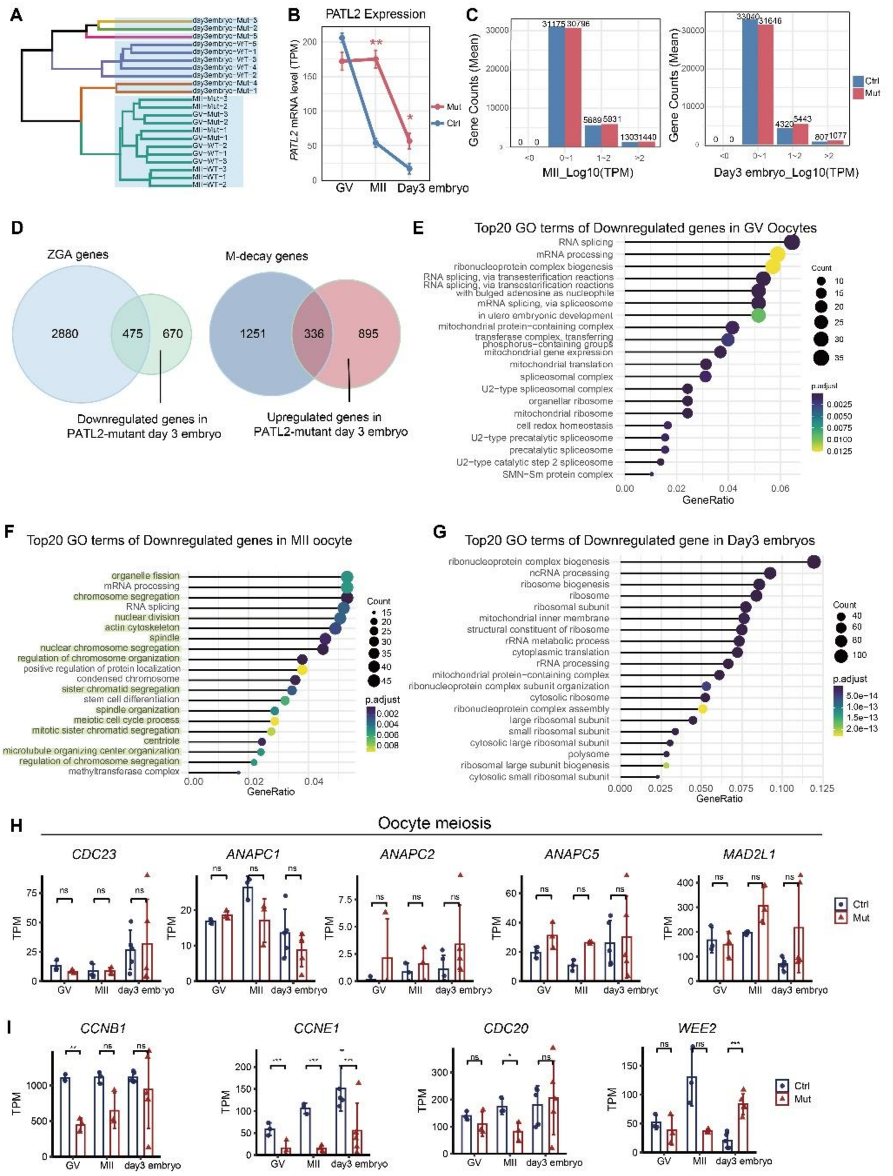

Figure S3. Comprehensive transcriptomic analysis of oocytes and early embryos harboring PATL2 mutations.

(A) Hierarchical clustering of samples indicating transcriptional profiles across control and mutant lines for germinal vesicle (GV) stage oocytes, metaphase II (MII) oocytes, and Day 3 embryos, with triplicates shown for clarity. **(B)** Line graph demonstrating the *PATL2* mRNA levels across developmental stages (GV, MII, Day 3 embryo). Data are expressed as mean ± SD using TPM. Statistical significance was determined using a *t*-test; $^{**}P < 0.01$, $^{*}P < 0.05$; ns, no significance. **(C)** Bar charts representing mean gene counts per sample across different developmental stages based on the transcript per million (TPM) categories. **(D)** Venn diagrams illustrating the distribution of zygotic genome activation (ZGA) genes and maternal-decay genes among upregulated and downregulated genes in Day 3 embryos from PATL2 mutants. **(E-G)** Bubble chart displaying the top 20 Gene Ontology (GO) terms associated with genes downregulated in GV oocytes (E), MII oocytes (F) and day 3 embryos (G). **(H-I)** Bar graph showcasing expression levels of selected genes (*CDC23*, *ANAPC1*, *ANAPC2*, *ANAPC5*, *MAD2L1*) in (H) and the selected differential expression genes (*CCNB1*, *CCNE1*, *CDC20* and *WEE2*) in (I) in RNA-seq involved in oocyte meiosis, compared between mutant and control groups at GV, MII, and Day 3 embryo stages, with statistical significance denoted. Data are expressed as mean ± SD using TPM. Statistical significance was determined using a t-test; $^{***}P < 0.001$, $^{**}P < 0.01$, $^{*}P < 0.05$; ns, no significance.

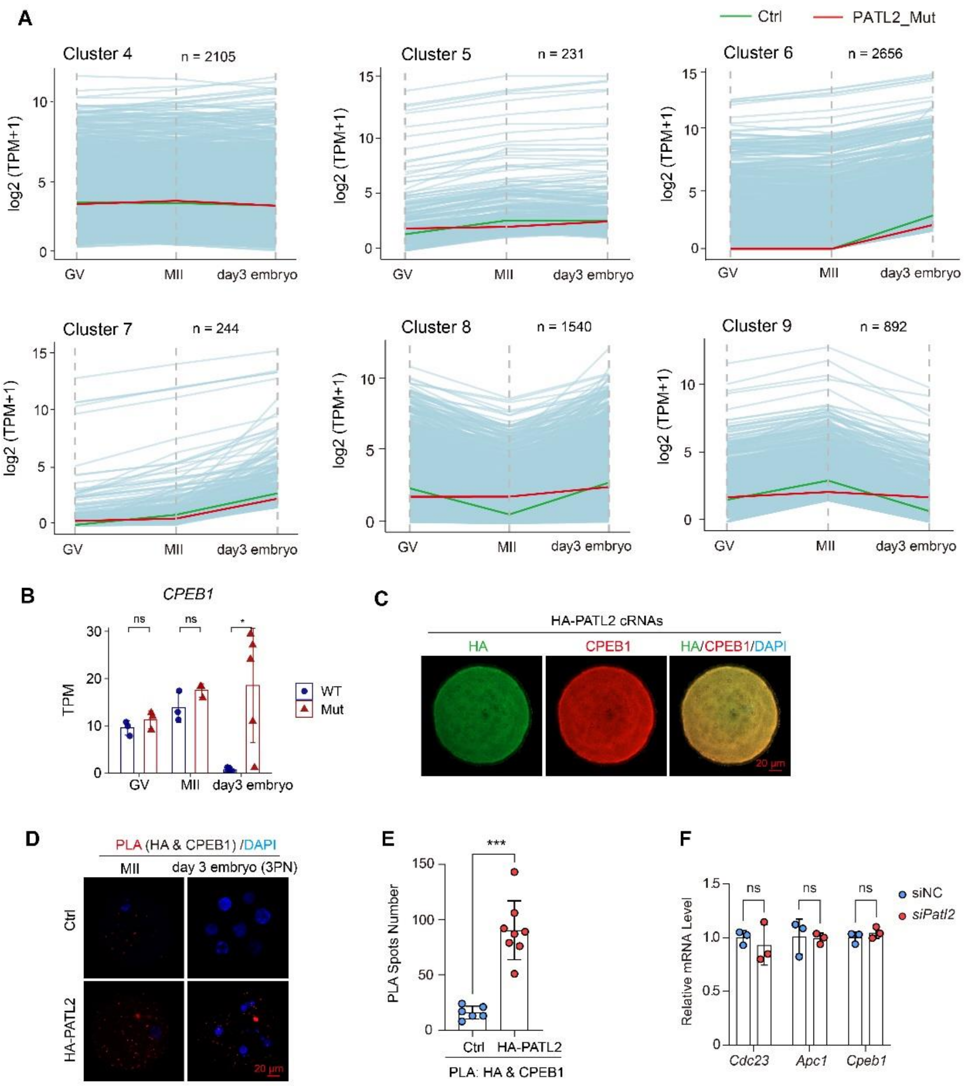

Figure S4. PATL2 interacts with CPEB1 and the effect of *Patl2* knockdown on oocyte maturation and *Cpeb1* expression.

(A) Transcripts patterns of human GV, MII oocytes and day 3 embryos. Each light blue line represents the expression levels of one gene. The green line and red line represent the median expression levels of the cluster in control and PATL2-mutant group, respectively. **(B)** Bar graph showing the mRNA expression level of *CPEB1* in RNA-seq compared between mutant and control groups at GV, MII, and Day 3 embryo stages. Data are expressed as mean ± SD using TPM. Statistical significance was determined using a *t*-test; **P* < 0.05; ns, no significance. **(C)** Representative immunofluorescence images showing the localization of HA-PATL2 and CPEB1 in human oocytes microinjected *HA-PATL2* cRNAs. Scale bars = 20 μm. **(D)** In situ proximity ligation assays (PLA) showing the interaction between HA-PATL2 and CPEB1 in human oocytes and 3PN embryos with or without microinjection of *HA-PATL2* cRNAs. Scale bar = 20 μm. **(E)** The graph showing the PLA spots numbers in (C). Data are expressed as mean±SD. Two-tailed Student's *t*-test; ****P*<0.001. **(F)** RT-qPCR results showing the *Cdc23, Apc1* and *Cpeb1* mRNA levels in MII oocytes with siNC and si*Patl2* knockdown (n= 3 biological replicates). Data are expressed as mean ±SD. Two-tailed Student's *t*-test; ns, no significance.

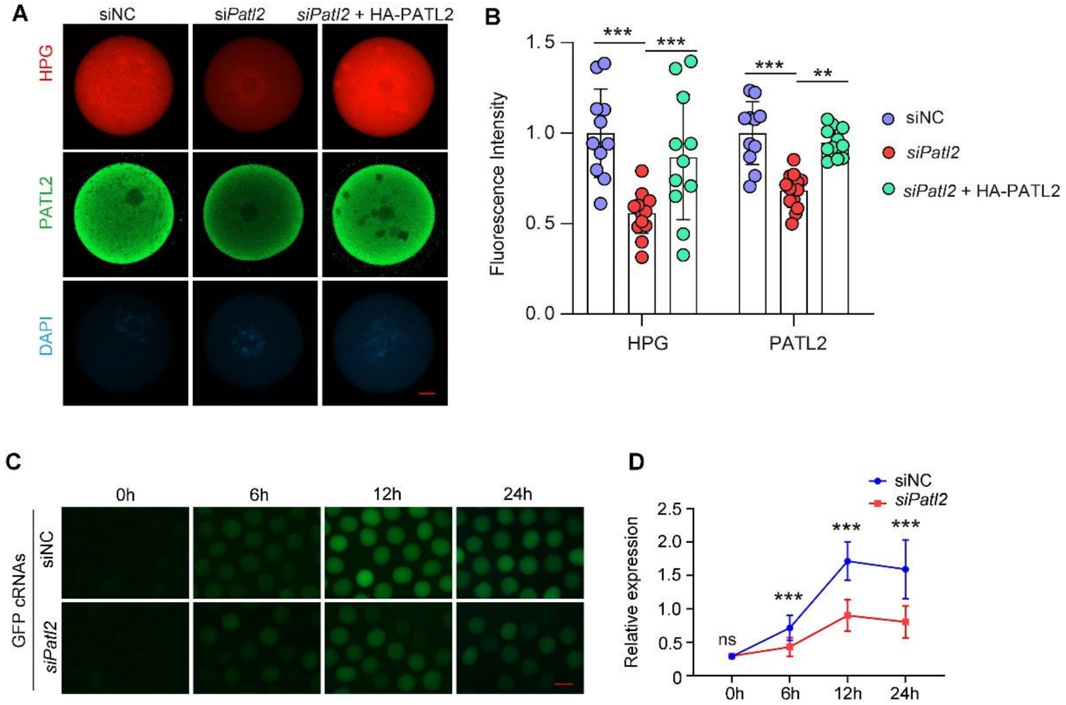

Figure S5. *Patl2* knockdown affects mRNA translation in mouse oocytes.

(A) HPG staining showed decreased protein synthesis in *Patl2*-knockdown oocytes and human PATL2 reversed the HPG intensity. GV oocytes were microinjected with siNC, siPatl2 or siPatl2 combination with HA-PTAL2. Scale bars= 10 µ. **(B)** The graphs showing the HPG intensity in (A). Data are expressed as mean±SD. Two-tailed unpaired Student's t-test. ***P*<0.01, ****P*<0.001. **(C)** (D) Representative images showing the GFP signal at different time points (0 h, 6 h, 12 h and 24 h) in siNC or *Patl2*-knockdown GV oocytes co-injection with GFP cRNAs. GV oocytes were microinjected with siNC or siPatl2 combination with GFP cRNAs. Scale bars= 100 µm. **(D)** The line graphs showing the GFP intensity in (C). Data are expressed as mean±SD. Two-tailed unpaired Student's t-test. ***P*<0.01; ****P*<0.001; ns, no significance.

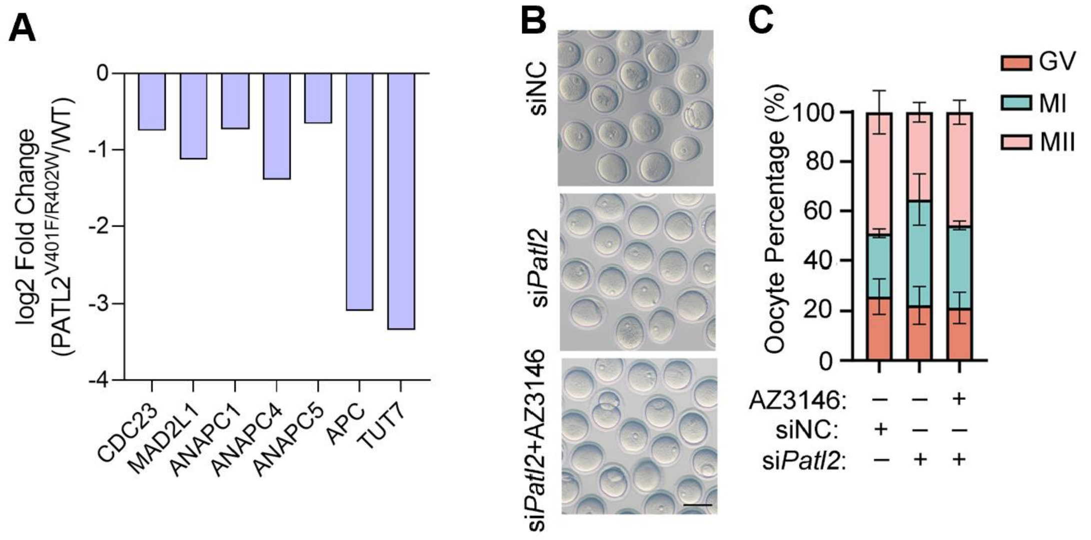

Figure S6. AZ3146 partially rescued the oocyte maturation defect induced by *Patl2* knockdown. (A) The bar graph showing the the PATL2$^{V401F/R402W}$ variant decreased binding intensity with CDC23, MAD2L1, ANAPC1 (APC1), ANAPC4 (APC4), ANAPC5 (APC5), APC and TUT7 after calibration with wild-type PATL2 intensity in MS/MS data. **(B)** The bright-field images showing the oocytes after microinjection of negative control or mouse *Patl2* siRNAs combined with or without 2 uM AZ3146 treatment during oocyte maturation. Three independent experiments were conducted (n >130 oocytes in each group). Scale bar = 100 µm. **(C)** The bar graphs showing the percentage of GV, MI and MII in (A).

Supplementary Tables

Table S1. Genomic PCR primers used to amplify the regions flanking the variants in the *PATL2* gene for Sanger sequencing.

**Region**	**F/R**[a]	**Primer Sequence (5' to 3')**	**PCR Size**
PATL2_exon 4	F	GGCCCAGCTGACTTTGAATC	356 bp
	R	CACACCCATACCTCACTTTGG	
PATL2_exon 12	F	AACCTGGAGTGAGTGTGGGA	408 bp
	R	ACCTTCTCTGGCATCTCTGG	
PATL2_exon 13	F	GAAGGGACTGTGGAAGCAAG	400 bp
	R	GACTGAGTGGGACCAAGGAG	
PATL2_exon 15-16	F	ATGCTCTCTAGGACAGACATGGT	339 bp
	R	GGTTGGCTGAAGACTTAGTCAAG	

[a]F represents forward primers, and R represents reverse primers.

Table S2. Primers used for plasmid mutagenesis of *PATL2* gene.

**Name**	**F/R**[a]	**Primer Sequence (5' to 3')**	**Application**
$PATL2^{V401F}$	F	CATCTGCCCCTCCTGTTCCGGAGGGATGTG	c.1201G>T
	R	CACATCCCTCCGGAACAGGAGGGGCAGATG	
$PATL2^{R402W}$	F	CTGCCCCTCCTGGTCTGGAGGGATGTGGCTG	c.1204C>T
	R	CAGCCACATCCCTCCAGACCAGGAGGGGCAG	
$PATL2^{V401F/R402W}$	F	CACTTGACCCTCCACGACTCCTCCAAGGACTTC	c.1201G>T & c.1204C>T
	R	GAAGTCCTTGGAGGAGTCGTGGAGGGTCAAGTG	
$PATL2^{E428Nfs*3}$	F	CACTTGACCCTCCACGACTCCTCCAAGGACTTC	c.1284Adel
	R	GAAGTCCTTGGAGGAGTCGTGGAGGGTCAAGTG	
$PATL2^{L424S}$	F	GCAAATGTATTAGTCACTCGACCCTCCACGAACTCCTC	c.1271T>C
	R	GAGGAGTTCGTGGAGGGTCGAGTGACTAATACATTTGC	
$PATL2^{F539Cfs*19}$	F	AGCTGGAGGCCAGGATGGAGTGTTTGCCTGGATTTACT	c.1613+2_1613+3insGT
	R	AGTAAATCCAGGCAAACACTCCATCCTGGCCTCCAGCT	

[a] F represents forward primers and R represents reverse primers

Table S3. Primers used for RT-PCR and siRNA sequences for RNAi.

Name	F/R[a]	Primer Sequence (5' to 3')
Patl2 (mouse)	F	CCAGTTGGAAGGCACACACTA
	R	CCAGTTGGAAGGCACACACTA
Cdc23 (mouse)	F	TTCTTCGGTAGTCTCGGTGG
	R	CCAACTCCGCAGACCATTT
Apc1 (mouse)	F	AGGGCAACGATGATTGCAG
	R	GTAACCTCCTGAAGAGATCCCA
Cpeb1 (mouse)	F	CCTCCCAGATGCAAATGGCTT
	R	GCGAGATCCTCTAACTGAGGGT
Tut7 (mouse)	F	TGGTCTGGGAATACACTGACA
	R	AACTCTAAAGCATAGAACCGCAG
Gapdh (mouse)	F	AGGTCGGTGTGAACGGATTTG
	R	TGTAGACCATGTAGTTGAGGTCA
siNC	F	UUCUCCGAACGUGUCACGU
si*Patl2* (mouse)	F	GAAGAGACAGGCAGACAAA
si*Patl2* (mouse)	F	GCAAGGACUAAUGUUGCUA
si*Patl2* (mouse)	F	GCAAGGACUAAUGUUGCUA

[a] F represents forward primers and R represents reverse primers
