# Supplementary material for: PATL2 mutations affect human oocyte maternal mRNA homeostasis and protein interactions in cell cycle regulation
Source: Cell Biosci. 2024 Dec 31;14:157. doi: 10.1186/s13578-024-01341-2 (PMC11686847; doi:10.1186/s13578-024-01341-2)

Supplementary Materials

Source data of WB in Figures

Figure 3B

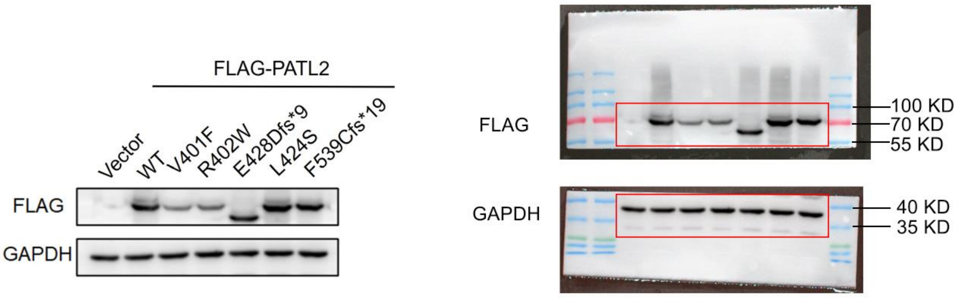

Figure 4G

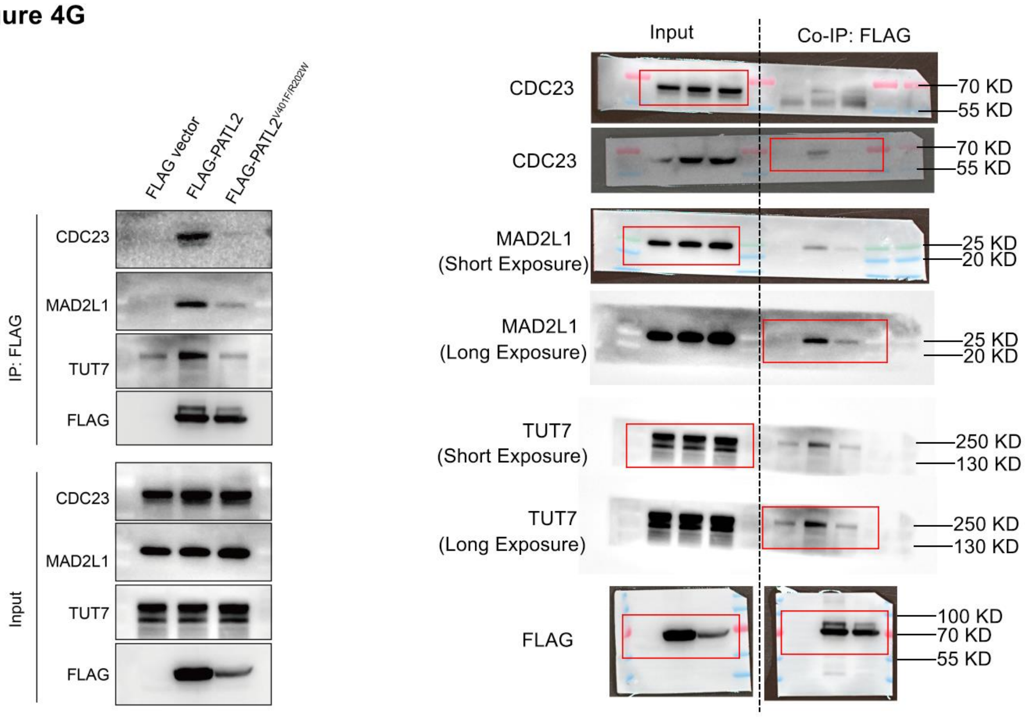

Figure 5A

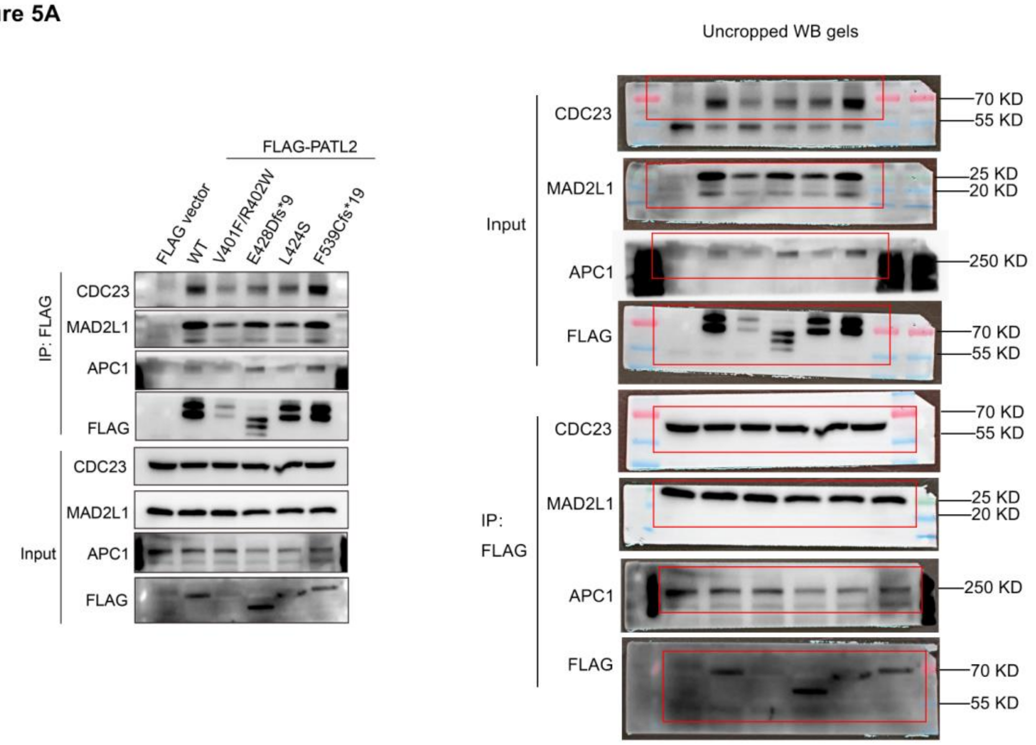

Figure 5B

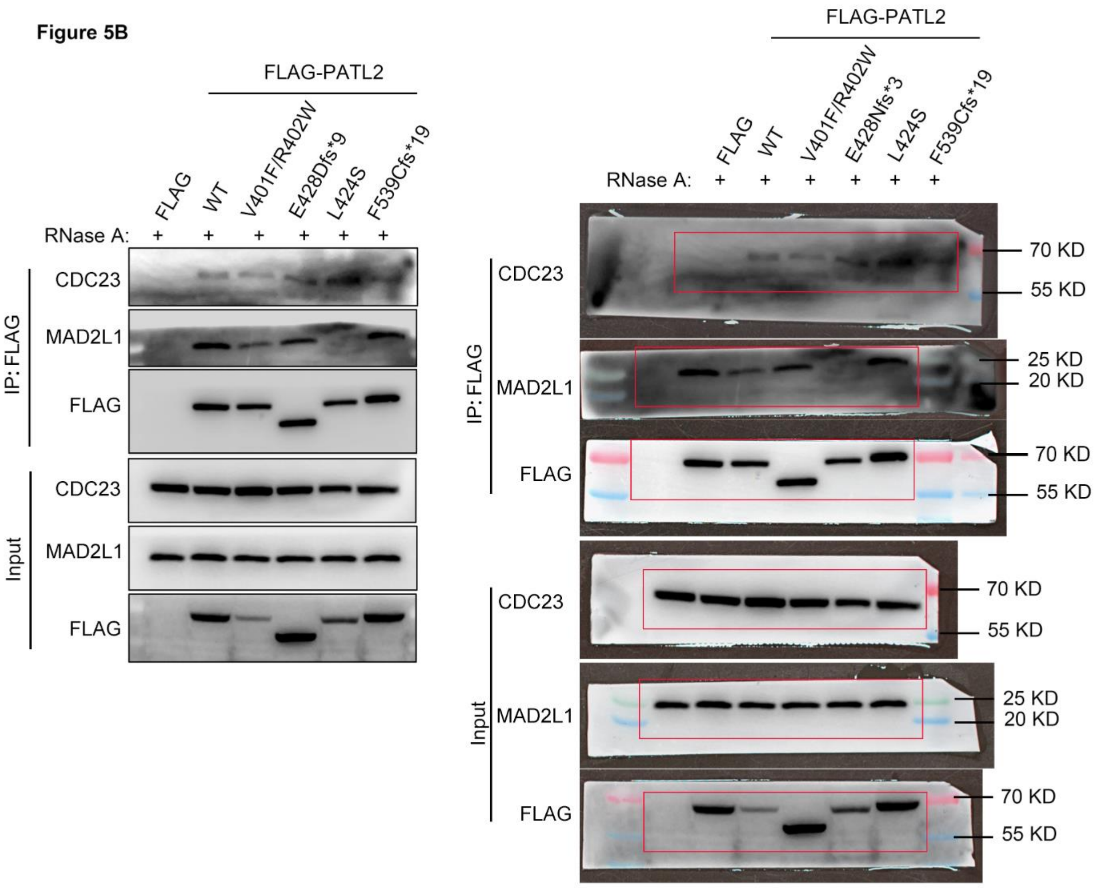

Figure 5C

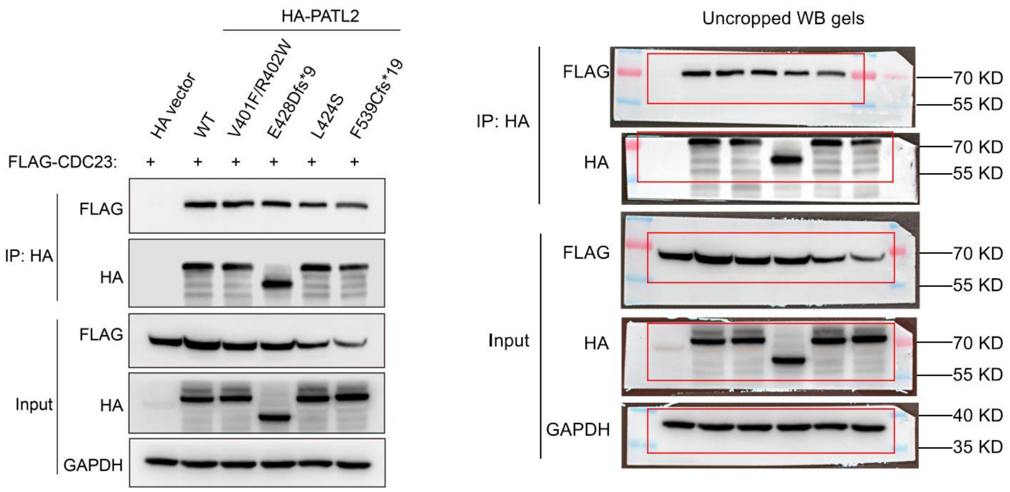

Figure 5I

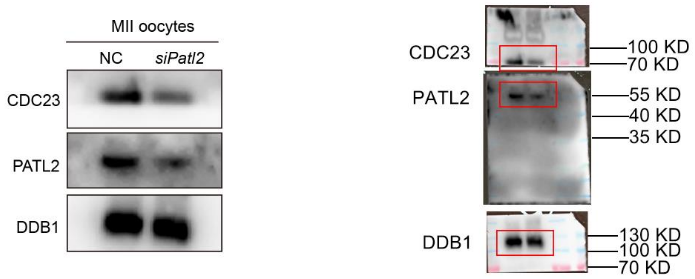

Figure 7F

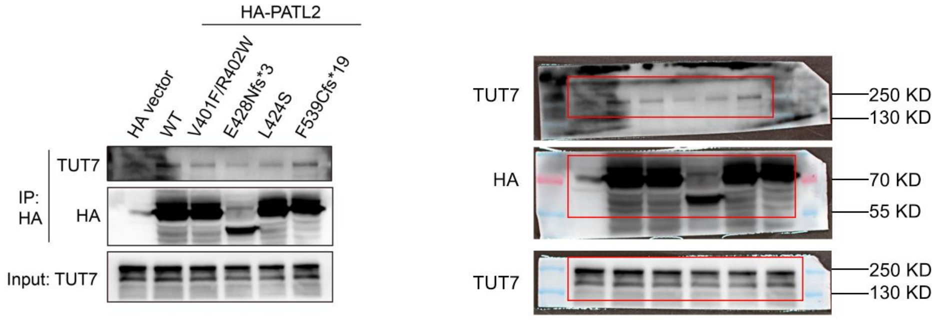

Supplement: Supplementary file 4 — Supplementary Material 4 [file 13578_2024_1341_MOESM4_ESM.pdf]
